# Structure of a Ty1 restriction factor reveals the molecular basis of transposition copy number control

**DOI:** 10.1038/s41467-021-25849-0

**Published:** 2021-09-22

**Authors:** Matthew A. Cottee, Sean L. Beckwith, Suzanne C. Letham, Sarah J. Kim, George R. Young, Jonathan P. Stoye, David J. Garfinkel, Ian A. Taylor

**Affiliations:** 1 Macromolecular Structure Laboratory, The Francis Crick Institute, London, UK; 2 Department of Biochemistry and Molecular Biology, University of Georgia, Athens, Georgia USA; 3 Bioinformatics and Biostatistics STP, The Francis Crick Institute, London, UK; 4 Retrovirus-Host Interactions Laboratory, The Francis Crick Institute, London, UK; 5 Department of Infectious Disease, Imperial College London, London, UK; 6 Present Address: Sir William Dunn School of Pathology, University of Oxford, Oxford, UK

**Keywords:** X-ray crystallography, Transposition

## Abstract

Excessive replication of *Saccharomyces cerevisiae* Ty1 retrotransposons is regulated by Copy Number Control, a process requiring the p22/p18 protein produced from a sub-genomic transcript initiated within Ty1 *GAG*. In retrotransposition, Gag performs the capsid functions required for replication and re-integration. To minimize genomic damage, p22/p18 interrupts virus-like particle function by interaction with Gag. Here, we present structural, biophysical and genetic analyses of p18m, a minimal fragment of Gag that restricts transposition. The 2.8 Å crystal structure of p18m reveals an all α-helical protein related to mammalian and insect ARC proteins. p18m retains the capacity to dimerise in solution and the crystal structures reveal two exclusive dimer interfaces. We probe our findings through biophysical analysis of interface mutants as well as Ty1 transposition and p18m restriction in vivo. Our data provide insight into Ty1 Gag structure and suggest how p22/p18 might function in restriction through a blocking-of-assembly mechanism.

## Abstract (web summary)

In *Saccharomyces cerevisiae*, unchecked proliferation of Ty1 retrotransposons is controlled by the process of copy number control (CNC), which requires the p22/p18 protein, translated from an internal transcript within the Ty1 GAG gene. Here, the authors present the 2.8 Å crystal structure of a minimal p18 from Ty1-Gag that is able to restrict Ty1 transposition and identify two dimer interfaces in p18, whose roles were probed by mutagenesis both in vitro and in vivo. As p22/p18 contains only one of two conserved domains required for retroelement Gag assembly, they propose that p22/p18-Gag interactions block the Ty1 virus-like particle assembly pathway, resulting in defective particles incapable of supporting retrotransposition.

## Introduction

Retrotransposons replicate through a reverse transcription step and are highly prevalent in eukaryotic genomes^1–3^. The budding yeast *S. cerevisiae* contains members of the Ty1-copia (Ty1, Ty2, Ty4, and Ty5), and Ty3-gypsy families of LTR retrotransposons^4^. Ty1 is the most abundant element in many strains with about 32 full-length copies in the reference strain S288C^5,6^. LTRs flank the 5′ and 3′ ends of the 5.9 kb genomic sequence that contains *GAG* and *POL* genes. After transcription from the 5′- to 3′- LTR, *GAG* encodes Gag-p49, which is analogous to retroviral Gag and provides both capsid (CA) packaging and nucleocapsid (NC) nucleic acid chaperone functions. *POL* encodes the protease (PR), integrase (IN), and reverse transcriptase (RT) enzymes, all of which are required for Ty1 replication and integration.

Ty1 replication and integration is similar to that of retroviruses but occurs intracellularly, and transposition is not infectious^7^. After transcription by RNA polymerase II, Ty1 genomic RNA is exported to the cytoplasm and two proteins are translated, Gag-p49 and Gag-Pol-p199, produced from +1 translational frameshift between the *GAG* and *POL* genes^8^. Gag-p49 and Gag-Pol-p199 assemble along with the incorporation of dimeric Ty1 genomic RNA^9^ at cytoplasmic foci called T-bodies or retrosomes to form virus-like particles (VLPs)^5,10–12^. Assembly of the immature VLP induces self-cleavage and release of PR, encoded in Gag-Pol-p199 and further cleavage of Gag-Pol-p199 then releases the IN and RT enzymes^5^. PR also cleaves Gag-p49 towards the C-terminus producing Gag-p45, the mature capsid and nucleic acid chaperone protein^13–15^, forming mature Ty1 VLPs, which perform similar functions to the viral core in retrovirus particles. Reverse transcription of Ty1 genomic RNA occurs within the VLP and the Ty1 cDNA-IN pre-integration complex is imported into the nucleus and integrated mainly at sites upstream of RNA polymerase III transcribed genes^16,17^.

Uncontrolled retrotransposition in the genome of any organism would be highly detrimental through the effects of integration in or near active genes causing mutation, unregulated expression, and genome instability^18–21^. Therefore, in higher eukaryotes mechanisms including RNAi pathways along with SAMHD1 and APOBEC restriction factors prevent excessive transposition^22,23^. In *S. cerevisiae*, these defence systems are not present and uncontrolled Ty1 retrotransposition is restricted by a separate mechanism, referred to as copy number control (CNC)^24–26^. A sub-genomic transcript initiated from within the Ty1 *GAG* gene, Ty1i, contains the C-terminal half of *GAG* as well as *POL*. A 22 kDa protein is translated from either of two alternative start codons, AUG1 or AUG2 found proximal to the 5′ end of the Ty1i transcript^25,27^. Thus, p22 is identical to the C-terminal half of Gag-p49 and is processed at the C-terminus by PR to generate a mature protein p18, that is identical to the C-terminal half of mature capsid Gag-p45^25,27^. Both p22 and p18 can restrict Ty1 retrotransposition, and so they constitute a self-encoded restriction factor. Although biochemical and fluorescence microscopy studies suggest that p22 associates with Gag-p45^25,27,28^, the mechanism of p22/p18 restriction of Ty1 retrotransposition is less clear. Multiple mechanisms have been proposed involving inhibition of different stages in the replication cycle^24^. These include inhibition of Gag-p45 nucleic acid chaperone function^27^, disruption of Ty1-Gag retrosome formation, and prevention of VLP assembly^25^.

Crystallographic and cryo-electron microscopy structural studies of retroviral Gag and CA have revealed how monomers first assemble into hexamers^29–31^, and when combined with CA pentamers can further assemble into closed Fullerene shell structures^32–37^ that are found in retroviral cores. The relative contribution from N- and C-terminal CA domains (NTD/CTD) in capsid assembly also varies in different retroviruses^29,38–40^. Structural studies of Ty3 Gag as well as the ARC protein from *Drosophila* (dARC) have revealed how the same hexameric and pentameric building blocks are utilised in VLP shell assembly^41–43^. However, to date no high-resolution structures of Ty1 Gag-p45 or p22/p18, or any member of the Ty1-Copia retrotransposon family are available to help define parameters of Ty1 VLP assembly or the structural basis of CNC.

Here we report the 2.8 Å crystal structure of a minimal p18 from Ty1-Gag (p18m) that is able to restrict Ty1 transposition. The structure comprises an all α-helical domain related to that observed in the CA-CTD of the yeast Ty3 retrotransposon, ARC proteins, and orthoretroviruses. The crystal structure contains two independent p18m dimer interfaces and analytical ultracentrifugation reveals a tight dimer that can further oligomerise. We test the significance of our structural findings using mutagenesis combined with biophysical studies in vitro and transposition and CNC analyses in vivo. Our work demonstrates the importance of an evolutionarily conserved transposon and retroviral CA-CTD interface and provides insight into a unified mechanism of Ty1 CNC.

## Results

### Defining a minimal Ty1 Gag p18 restriction domain

**Fig. 1:**
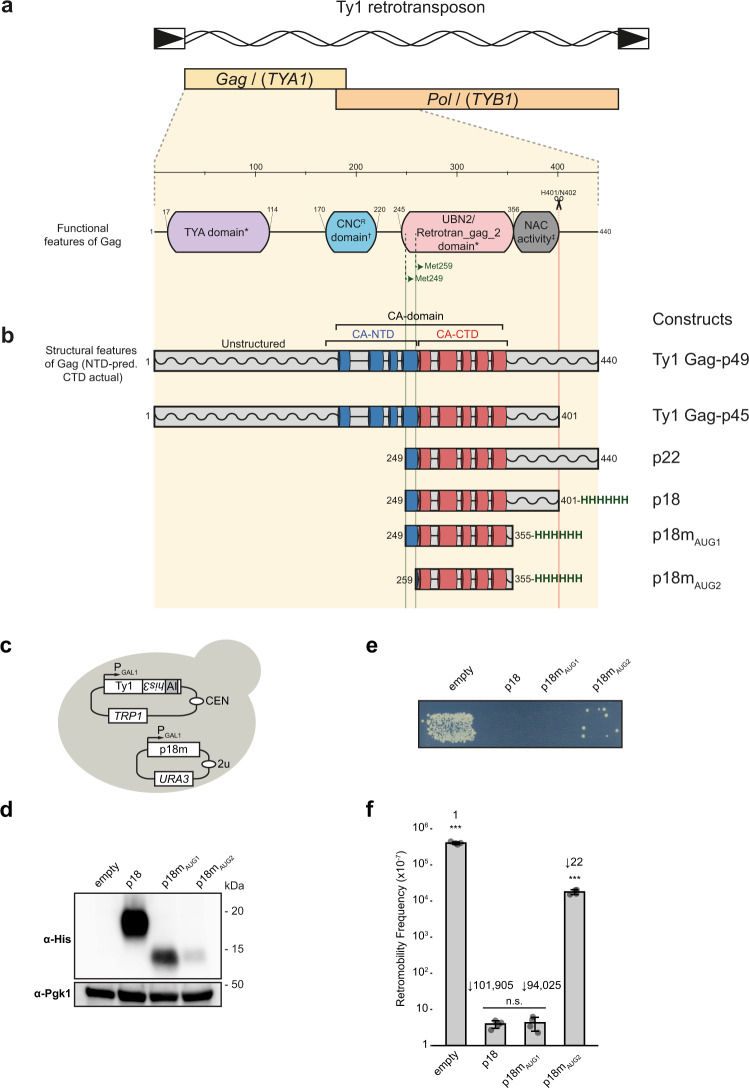
Transposition assays define the minimal fragment for p22/p18 activity. **a** Schematic of the Ty1 retrotransposon, highlighting the *TY1A GAG* and *TY1B POL* genes. The functional regions and cleavage site of Gag identified by genetic analysis are highlighted below, *PFAM domains, ^†^CNC^R^ domain^28^, ^‡^NAC originally defined as residues 299-401^15^ redefined by this study as residues 356–401. The Met249 and Met259 translational starts that initiate from the AUG1 and AUG2 codons are indicated. **b** Schematic of p22/p18 constructs and structural features of Gag. Gag-p49 and p22 undergo proteolytic maturation into Gag-p45 and p18, respectively. A restrictive fragment terminating at residue 355 (p18m) is defined in this study and all p18 constructs also contain a C-terminal hexa-histidine tag. **c** Schematic illustrating the two plasmids used to co-express the Ty1 transposon and the p18 restriction factor. The expression of each is driven by a galactose-inducible promoter from the *GAL1* gene. Ty1 contains the *his3-AI* retromobility indicator gene; histidine prototrophy requires retromobility. **d** Western blot of p18 fragment expression. Protein extracts of galactose-induced cells were immunoblotted with an anti-hexa-histidine antibody. Pgk1 serves as a loading control. Strains used: empty (DG3739), p18 (DG4162), p18m_AUG1_ (DG4147), p18m_AUG2_ (DG4146). Migration of molecular weight standards is shown alongside the immunoblot. A representative image of at least 3 replicates is shown, images of entire gel immunoblots are provided in the Source Data file. **e** Qualitative mobility assay showing CNC effect of p18 constructs on Ty1 retromobility. Cells were galactose-induced; growth on selective media lacking histidine indicates a retromobility event. A representative image of at least 3 replicates is shown. **f** Quantitative mobility assay of galactose-induced cells. Each bar represents the mean of the four independent measurements displayed as points. The error bar centre represents the mean of the four measurements and the error bar extent ± the standard deviation. Fold-change compared with empty vector is indicated above the bars. Significance is calculated from a two-sided Student’s *t*-test compared with p18 (n.s not significant, ***p < 0.001. Exact *p*-values are provided in Supplementary Table 1, source data is provided in the Source Data file.

Ty1 *GAG* contains several regions based on structure predictions, biochemical and genetic analyses, and phylogenomic comparisons (Fig. 1a). In particular, predicted helical regions, are contained within the CNC-resistance (CNC^R^) and UBN2/Retrotran_gag_2 PFAM domains, respectively^28^. The helical regions also correspond to the CA-NTD and CA-CTD of LTR retrotransposons and retroviruses^41^. Specific CNC^R^/CA-NTD amino acid substitutions confer resistance to the p22 restriction factor, and UBN2/CA-CTD is within p22. CNC^R^ mutations also occur in the UBN2/CA-CTD domain^28^.

Segments within the p22 and p18 coding sequence (Fig. 1b) were expressed ectopically and assessed for their capacity to restrict Ty1 retromobility. The constructs (Fig. 1b) were chosen based on the previously defined AUG1 and AUG2 initiation codons and on analysis of secondary structure predictions for Ty1-Gag^25,27,28^. Constructs also contained a hexa-histidine tag to aid in protein detection and purification. p18 constructs were inducibly co-expressed with a Ty1 element containing the *his3*-AI indicator gene^44^ to determine the effect on retromobility (Fig. 1c). All p18 deletion constructs were also assessed by Western blot (Fig. 1d). As expected, initiation at AUG1 (p18m_AUG1_) resulted in much higher levels of expression in yeast than initiation at AUG2 (p18m_AUG2_)^27,45^. Constructs were tested for their capacity to inhibit Ty1 mobility in a qualitative plating assay, and these data showed that truncated tagged p18 proteins were still able to restrict Ty1 transposition (Fig. 1e). Quantitative mobility assays (Fig. 1f and Supplementary Table 1) revealed that a fragment containing AUG1 to residue 355 of p18 retained potent restriction of Ty1, comparable to full-length p18. The restriction is also apparent with p18m_AUG2_ but at a lower level, correlating with the reduced expression. The co-expression results show that regions of Gag required for CNC comprise residues M249-N355 in p18m_AUG1_ or M259-N355 in p18m_AUG2_ and imply that the nucleic acid chaperone domain of Gag (Fig. 1a) is not required for CNC.

### Structure of the Ty1 Gag p18m domain

**Fig. 2:**
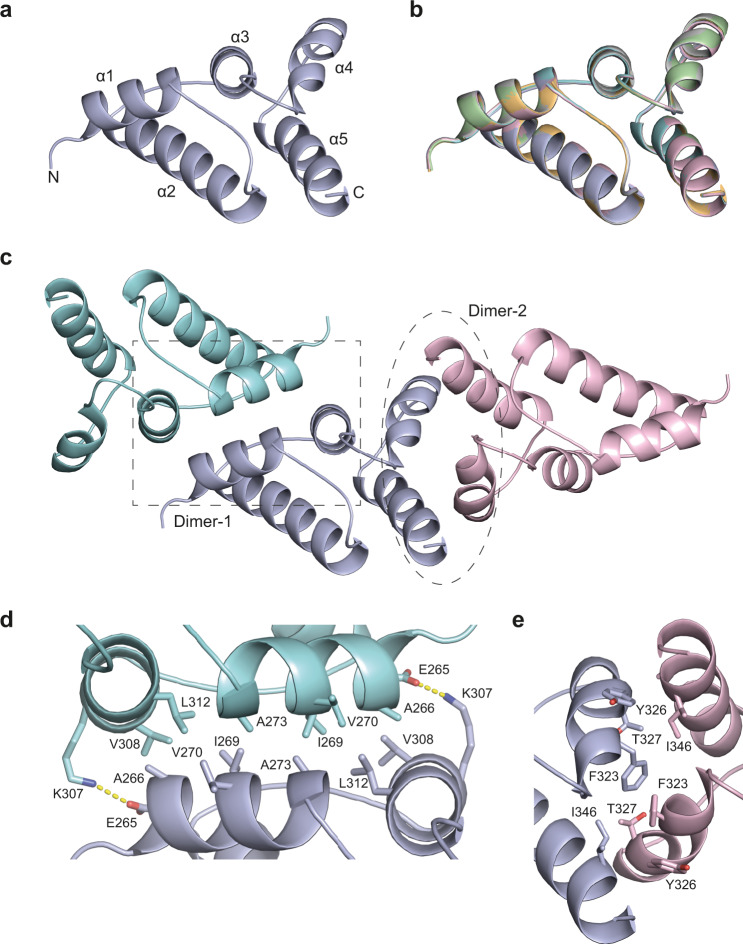
Crystal structure of p18m. **a** Crystal structure of p18m monomer, the protein backbone is shown in cartoon representation. The five α-helices in the structure are labelled sequentially from the N- to C-terminus. **b** 3D structural alignment of all six copies of p18m present in the AUG1 and AUG2 structures. The view is the same as in **a**. Individual monomers are shown in cartoon representation coloured light-blue, cyan, pink, orange, grey, and green. Structures were aligned using backbone Cα atoms (RMSD 0.16 ± 0.04 Å over 82 Cα). **c** The trimer asymmetric unit of the p18m crystal structure. The three p18m protomers are shown in cartoon representation coloured cyan, light-blue and pink respectively. The dimer interfaces between protomers (Dimer-1 and Dimer-2) are highlighted with the grey box and ellipse respectively. **d** Close-up view of the Dimer-1 interface, the box-highlighted region in (**c**). Residues on helices α1 and α3 that make apolar and salt bridge interactions are shown in stick representation. The E265 and K307 salt bridge is indicated by the dashed lines. **e** Close-up view of the Dimer-2 interface, the ellipse-highlighted region in (**c**). Residues on helices α4 and α5 that make the hydrophobic interactions are shown in stick representation.

We determined the crystal structures of p18m_AUG1_ and p18m_AUG2_ expressed in *E. coli*. The p18m_AUG2_ structure was solved by multi-wavelength anomalous diffraction (MAD) using crystals of Se-Met substituted protein. The p18m_AUG1_ structure was solved by molecular replacement using the p18m_AUG2_ structure as a search model. Both constructs crystallised in the same P6_5_22 hexagonal spacegroup with the p18m_AUG1_ crystals diffracting to a slightly higher resolution of 2.8 Å and the structure refined to an R-factor and Free R-factor of 25.6 and 26.4% respectively (Supplementary Table 2). In both structures, the asymmetric unit (ASU) comprises three copies of the p18m monomer. The structure has an all-helical fold making a five-helix bundle (α1-α5) comprising α1 (residues E265-A273), α2 (residues D284-N300), α3 residues (N306-M314), α4 (residues Y321-R330) and α5 (residues V336-Q351) (Fig. 2a). In all copies, residues spanning D262 to Q351 are visible in the electron density map. The structural superposition of all six copies, three from the p18m_AUG1_ structure and three from the p18m_AUG2_ structure (Fig. 2b), have an RMSD of <0.25 Å for all pairwise alignments overall Cα atom positions. There is no additional density for residues 249 to 259 that constitute the additional N-terminal sequence difference between AUG1 and AUG2. Therefore, we consider both structures to be identical, and define p18m as residues M259-N355 (p18m_AUG2_) and representing the minimal domain required for CNC activity.

### Structural similarity with ARC and retroviral CA

Structural similarity searches of p18m using the DALI search engine^46^ identified *Drosophila* and mammalian ARC CA-CTD structures as top hits with DALI Z scores ranging from 8.6–7.0 (Supplementary Fig. 1). The CTD of Ty3 CA also provided a strong match with a DALI Z score of 7.7. These topological and structural similarities reflect the evolutionary relationship between retrotransposons and the exapted ARC proteins. There were also matches with CA-NTDs from ARC proteins (DALI Z score 5.8) and Ty3 (DALI Z score 6.1), supporting the notion that tandem domains of CA arose as the result of a gene duplication event^47^. The closest matching retroviral structure was with the CA-CTD from the gamma-retrovirus MLV with a DALI Z score of 7.8. Weaker matches with the CA-CTD and CA-NTD from HIV-1 and the CA-CTD from the endogenous retrovirus HERV-K (DALI Z-scores 5.3–4.9) demonstrate the more distant relationship between retrotransposon and retroviral CAs.

### The p18m dimer interfaces

Inspection of the p18m crystal structures revealed three monomers in the ASU, each forming two dimer interfaces (Fig. 2c). In the first (Dimer-1), the exposed surfaces of α1 and α3 pack against α1’ and α3’ of the opposing monomer (Fig. 2d). The entire dimer interface encompasses 773 Å^2^ of buried surface and is defined by largely hydrophobic interactions with contributions from sidechain packing of A266, I269, V270, A273 on α1 and I302, I304, V308, and L312 on α3 and the preceding interspersing α2-α3 loop that form a continuous apolar network with I269 and A273 at its centre (Fig. 2d). In addition, E265 and K307 at the N-termini of α1 and α3, respectively, make a salt bridge interaction at the periphery of the interface that further stabilises the dimer. The extent and hydrophobic nature of interactions within this homodimer interface suggests the dimer constitutes a relatively stable or obligate structure. Moreover, this interface is conserved amongst dARC and retroviral CA-CTD structures (Supplementary Figure 2) that also comprise an equivalent hydrophobic core.

To analyse surface conservation at this interface, we conducted a multiple sequence alignment of 125 Ty1 Gag sequences found in *Saccharomyces* genomes. The alignment (Supplementary Fig. 3a) was mapped onto the structure using the Consurf server^48,49^ and revealed that residues making significant contributions to the interface (especially the highly hydrophobic patch formed by I269/I302/I304/V308 and salt-bridging residues K307/E265) were near-universally conserved (Supplementary Fig. 3b, c). Other interface residues were substituted for similar residues. Our analyses suggest that this hydrophobic dimerisation interface is a conserved feature of Ty1 Gag found throughout *Saccharomyces* and is similar in nature to CTD dimers from divergent CA proteins.

In the second interface (Dimer-2), residues on the outer surface of α4 and α5 pack against α4′ and α5′ of the opposing monomer (Fig. 2e). This dimer interface encompassed 690 Å^2^ of buried surface and comprises a hydrophobic network with sidechain packing of F323, Y326, and T327 on α4 with A345 and I346 on α5. Notably, the Dimer-2 interface is not sequence conserved in Ty3, dARC, or retroviral CA. However, upon structural superposition of p18m with Ty3 CA in assembled shells^41^ (PDB ID 6R24; DALI Z score 7.7), it is apparent that not only do the α4 and α5 helices of p18m and those of the Ty3 CA-CTD align well at the level of tertiary structure, they are located at the local inter-pentamer and inter-hexamer 3-fold axes in Ty3 (Supplementary Fig. 4) and by inference at the equivalent 3-fold axes of Ty1 particles^50^. These similarities raise the possibility that Dimer-2 in the p18m crystal structures is a remnant of the trimer formed in Ty1 CA assembled shells but that the crystal packing in this case does not allow for the formation of this trimer interaction.

### p18m self-associates in solution

**Fig. 3:**
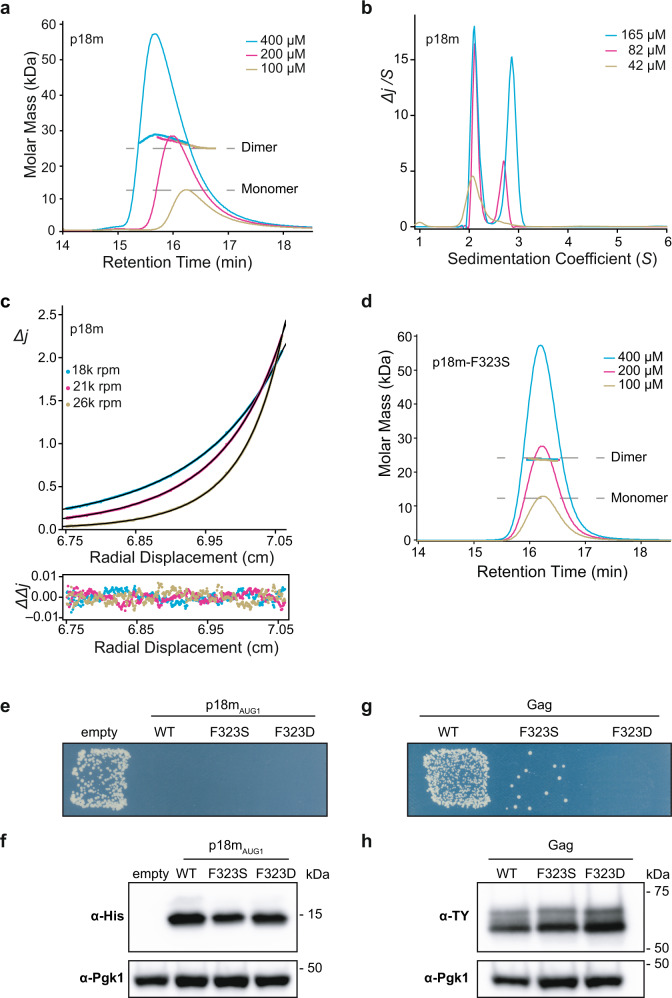
p18m self-associates in solution. **a**, **d** SEC-MALLS analysis of p18m and Dimer-2 interface-mutant p18m-F323S. The sample loading concentrations were 400 µM (cyan), 200 µM (magenta), and 100 µM (wheat). The differential refractive index (dRI) is plotted against column retention time and the molar mass, determined at 1 s intervals throughout the elution of each peak, is plotted as points. The p18m monomer and dimer molecular mass is indicated with the grey dashed lines. **b** C(S) distributions were derived from sedimentation velocity data recorded from p18m at 42 µM (wheat), 82 µM (magenta), and 165 µM (cyan). The curves are the distribution of the sedimentation coefficients that best fit the sedimentation data (RMSD 0.004−0.016), see also Supplementary Table 3. **c** Multispeed sedimentation equilibrium profiles determined from interference data collected on p18m at 30 µM. Data were recorded at the speeds indicated. The solid lines represent the global best fit to the data using a monomer-dimer-tetramer model (*K*_*D*_^*(1-2)*^ = 0.73 µM, *K*_*D*_^*(2-4)*^ = 43.2 µM; reduced *χ*^*2*^ = 2.22). The lower panel shows the residuals to the fit, see also Supplementary Table 3. Source data for **b** and **c** are provided in the Source Data file. **e**, **g** Effect of p18m_AUG1_ and Gag mutations on Ty1 mobility. Growth on selective media; His^+^ cells indicate a retromobility event. A representative image of at least 3 replicates is shown. p18m_AUG1_ strains used: empty (DG3739), WT (DG4147), F323S (DG4350), F323D (DG4351). Gag strains used: WT (DG3735), F323S (DG4348), F323D (DG4349). **f**, **h** Protein extracts prepared from galactose-induced yeast cells expressing the indicated p18m_AUG1_ or Gag mutants were immunoblotted with hexa-histidine antibody to detect p18m_AUG1_ or TY-tag antibody to detect Gag. Pgk1 serves as a loading control. Migration of molecular weight standards is shown alongside the immunoblots. A representative image of at least 3 replicates is shown. Images of the whole gel immunoblots are provided in the Source Data file.

Given two different dimer interfaces were observed in the p18m crystal structure, the solution molecular mass, conformation, and self-association properties of p18m were examined using solution hydrodynamic methods. Initial Size Exclusion Chromatography coupled Multi-Angle Laser Light Scattering (SEC-MALLS) analysis was performed with protein concentrations ranging from 100–400 µM. These data yielded a solution molecular weight of 26 kDa for p18m but with an indication of the further weak association at the highest concentrations employed (Fig. 3a). The p18m sequence-derived molecular weight is 12.2 kDa. Therefore, p18m forms strong dimers in solution with some tendency for further self-association at higher concentrations.

To further understand p18m oligomerisation, sedimentation velocity (SV-AUC), and sedimentation equilibrium (SE-AUC) analytical ultracentrifugation were employed to analyse p18m hydrodynamic properties (Supplementary Table 3). Sedimentation velocity data for p18m over a concentration range of 42–165 µM and analysed using the C(S) continuous sedimentation coefficient distribution function revealed two predominant species (Fig. 3b). All p18m samples contained a slow component with *S*_*20,w*_ of 2.32 ± 0.03. However, at increasing protein concentration a fast component was detected with a sedimentation coefficient that increased from *S*_*20,w*_ of 2.90 at 82 µM to *S*_*20,w*_ of 3.09 at 165 µM. Concentration dependency was also apparent by evaluation of the weight average sedimentation coefficient, obtained by integration of the entire envelope of the C(S) function, which also showed an increase with increasing concentration (Supplementary Table 3).

The solution molecular weight of the slow and fast components was determined by combining the sedimentation coefficients with the best fit frictional ratio (*ƒ*/*ƒ*_*0*_) from the C(S) analysis. This gave 21.9 ± 0.7 kDa for the slow species, close to the formula mass of a p18m dimer, and a concentration-dependent value of 30.0–33.7 kDa for the fast species. These data show that p18m comprises a stable 2.32 *S* species with a molecular weight consistent with a p18m dimer, but that this dimeric species can also further self-associate into larger oligomers, consistent with the behaviour observed by MALLS. To further characterise p18m self-association, multispeed SE-AUC studies at varying protein concentrations were carried out. Typical equilibrium distributions recorded at the three speeds are presented in Fig. 3c. Analysis of individual gradient profiles using a simple individual species model showed there was a strong concentration dependency of the molecular weight ranging from 31.6 to 39.6 kDa and poor fitting of the data. Given our observations in the sedimentation velocity experiments, the data were fitted globally using a monomer-dimer-tetramer model (Fig. 3c and Supplementary Table 3). The application of this model gave a much-improved best fit that comprised p18m monomers in a tightly associating monomer-dimer equilibrium, *K*_*D*_^*(1-2)*^ of 0.73 µM, together with a weakly associating dimer-tetramer equilibrium, *K*_*D*_^*(2-4)*^ of 43.2 µM.

### Dimer-2 is required for transposition but not restriction

To test the functional significance of p18m self-association, we first introduced an F323S mutation at the Dimer-2 interface to disrupt α4-α4 hydrophobic interactions. The introduction of this polar sidechain had minimal effects on protein expression. Assessment of the p18m-F323S solution oligomeric state by SEC-MALLS yielded a solution molecular weight of 26 kDa and showed that further higher-order association was suppressed over the concentration range tested (100–400 µM) (Fig. 3d). This suggests that the Dimer-2 interface mediates only the weak higher-order association whilst the Dimer-1 interface is responsible for forming the strong dimer we observe in the solution.

In yeast, disruption of the Dimer-2 interface by the F323S or more severe charge-clash F323D mutation in p18m_AUG1_ did not markedly impair restriction or reduce protein expression (Fig. 3e, f), supporting the notion that the Dimer-2 interface can be modified and p18m still retain function in vivo. By contrast, whilst the introduction of F323S or F323D into Ty1 *GAG* did not affect Gag expression compared with WT, both substitutions dramatically reduced retromobility with F323D having the severest effect (Fig. 3g, h).

### Dimer-1 Gag assembly mutations affect Ty1 mobility and CNC

We investigated the functional significance of the Dimer-1 hydrophobic interface by targeted mutagenesis. We first made interface-disruptive mutations that were polar I269S and A273Q, charged I269K and A273D, or increased hydrophobic-bulk, A273M. All resulted in the loss of protein stability/solubility, as judged by our inability to recover and purify soluble proteins when expressed in *E. coli*. Additionally, the introduction of I269S or A273M mutations in either p18m or Gag dramatically reduced protein accumulation in yeast (Supplementary Table 4).

**Fig. 4:**
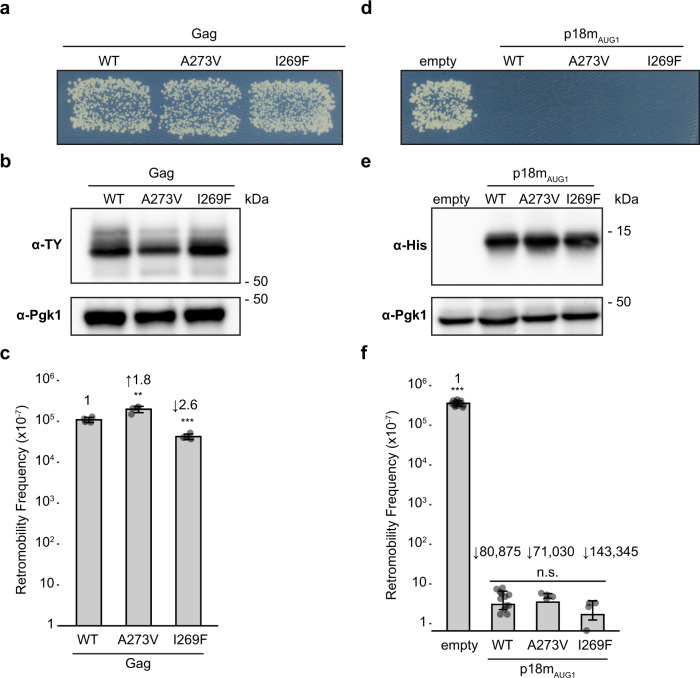
Ty1 retromobility and restriction tolerates conservative interface mutants. **a**, **d** Effect of Gag and p18m_AUG1_ mutations on Ty1 mobility. Growth on media lacking histidine indicates a retromobility event. A representative image of at least 3 replicates is shown. Gag strains used: WT (DG3735), A273V (DG4342), I269F (DG4341). p18m_AUG1_ strains used: empty (DG3739), WT (DG4147), A273V (DG4165), I269F (DG4340). **b**, **e** Protein extracts prepared from galactose-induced yeast cells expressing the indicated Gag or p18m_AUG1_ mutants were immunoblotted with TY-tag antibody to detect Gag or hexa-histidine antibody to detect p18m_AUG1_. Pgk1 serves as a loading control. Migration of molecular weight standards is shown alongside the immunoblots. A representative image of at least 3 replicates is shown, Images of the whole gel immunoblots are provided in the Source Data file. **c**, **f** Quantitative mobility assay of galactose-induced cells. Each bar represents the mean of at least four independent measurements displayed as points. The error bar centre represents the mean of the measurements and the error bar extent ± the standard deviation. Fold-change compared with WT is indicated above the bars. Significance is calculated from a two-sided Student’s *t*-test compared with WT (n.s not significant, **p < 0.01, ***p < 0.001. Exact *p*-values are provided in Supplementary Table 1, See also Supplementary Table 4. Source data is provided in the Source Data file.

We also made random mutations at the key Dimer-1 interface residues I269 and A273 and characterised them in the context of *GAG* within a complete Ty1 element. An NNK mutagenesis strategy^51^ (see Methods) was applied to randomize all 20 amino acid codons at I269 and A273 while reducing premature stop codons. Results from both NNK mutagenesis (Supplementary Fig. 5) and targeted mutagenesis (Supplementary Table 4) fit the trend that like-for-like mutations are tolerated but non-conservative mutations interfere with Gag accumulation and transposition. Of the tolerated conservative substitutions, we characterized the effects of A273V, previously identified as an escape mutant from p22-based CNC^28^, and I269F. In qualitative plate assays, galactose-induced expression of Ty1 Gag-A273V and Gag-I269F showed levels of transposition that were indistinguishable from WT Gag (Fig. 4a) and expressed at similar levels (Fig. 4b). Quantitative mobility assays revealed only small changes in Ty1 movement (Fig. 4c and Supplementary Table 1), indicating Gag-A273V and Gag-I269F retain the capacity to support transposition.

We characterised Dimer-1 interface mutations A273V and I269F in the context of p18m_AUG1_ to examine their ability to restrict WT Ty1. The p18m-A273V and p18m-I269F mutants were well-expressed as monitored by inducible co-expression of p18m and Ty1 and strongly inhibited Ty1 mobility in qualitative assays (Fig. 4d, e). They restricted Ty1 transposition by 10^5^-fold, indistinguishable from that of p18m (Fig. 4f and Supplementary Table 1). These data further support the idea that the p18m Dimer-1 interface is a requirement for protein folding/structural integrity and likely forms a key building block of Ty1 particle assembly. As a result, only conservative mutations that do not perturb the Dimer-1 hydrophobic network are tolerated and able to retain both p18m and Gag functionality.

### Self-association and stability of p18m interface mutants

**Fig. 5:**
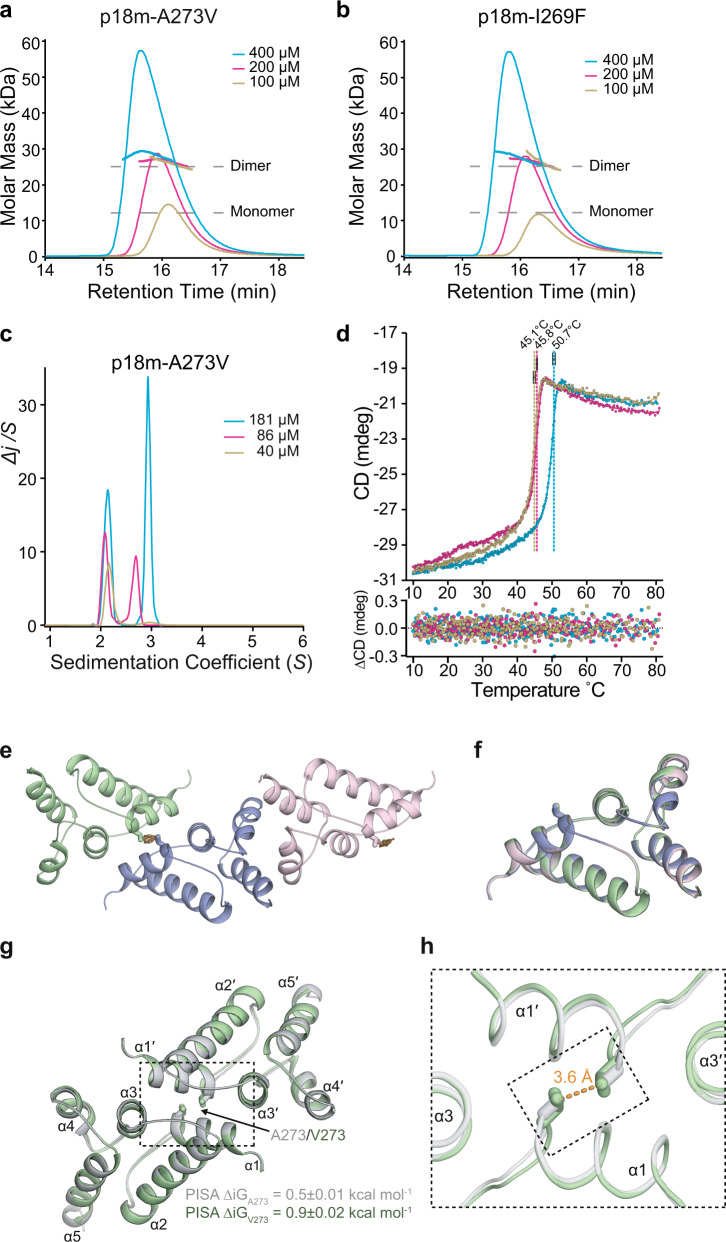
p18m Dimer-1 interface mutants assembly and structure. **a**, **b** SEC-MALLS analysis of p18m-A273V and p18m-I269F, sample loading concentrations; 400 µM (cyan), 200 µM (magenta), and 100 µM (wheat). dRI is plotted against retention time. The molar mass, determined at 1 s intervals throughout peak elution, is plotted as points. Monomer and dimer molar masses are indicated with the dashed lines. **c** C(S) distributions were derived from sedimentation velocity data recorded from p18m-A273V at 40 µM (wheat), 86 µM (magenta), and 181 µM (cyan). Curves are the distribution of the sedimentation coefficients that best fit the sedimentation data (RMSD 0.009-0.025), see also Supplementary Table 3. (**d**) Thermal denaturation of p18m, p18m-A273V, and p18m-I269F monitored by CD at 222 nm upon heating from 10–80 °C. Curves are the best fit spline function to the data points, dashed lines indicate the *T*_*m*_ of transitions determined from the 1st derivative of the fitted curves. Error bars are standard deviations from three independent measurements. Data shown are from one representative experiment, the lower panel shows the residuals to each fit. Source data for **c** and **d** is provided in the Source Data file (**e**) Asymmetric unit of the p18m-A273V crystal structure. The backbone of the three p18m protomers are shown in the cartoon, coloured lime, slate and pink. The Fo-Fc map contoured at 3σ (orange mesh), produced after molecular replacement, contains residual positive density for the valine γ-methyl groups at residue 273. **f** 3D superimposition of the 3 chains shown in (**e**). Structures were aligned using backbone Cα atoms (rmsd = 0.16 ± 0.04 Å over 78 ± 3.6 Cα). **g** Comparison of p18m (grey) and p18m-A273V (green) dimer interfaces. Structures are shown in cartoons with A273 or V273 sidechains as sticks. The PISA-calculated average solvation energy contribution to each dimer from either A273 (p18m) or V273 (p18m-A273V) are shown below. **h** Close-up view of the dimer interface, boxed in **g**. Local shifts in the backbone conformation at the C-terminus of α1 prevent steric clashes and allow the packing of the additional γ-methyl groups of V273 preserving a favourable 3.6 Å Van der Waals distance.

Since the I269F and A273V mutants were tolerated in vivo, p18m_AUG2_ mutants were examined for their effects on protein oligomerisation and stability. A SEC-MALLS analysis performed at increasing protein concentration (100–400 µM) yielded a molecular weight of 26 kDa for both p18m-A273V and p18m-I269F, consistent with the dimer molecular weight. Further weak higher-order self-association at the highest concentrations employed was also evident (Fig. 5a, b). These data demonstrate that the I269F or A273V mutations are accommodated within the interface without disrupting p18m dimerisation.

To characterise the assembly properties of the I269F and A273V mutants and quantify the affinity of self-association interactions, SV- and SE-AUC measurements were undertaken (Supplementary Table 3). The best fit C(S) functions were determined from SV-AUC data recorded from p18m-A273V and p18m-I269F over a concentration range of 30–180 µM (Fig. 5c and Supplementary Fig. 6a). For both interface mutants, as was observed with p18m, the C(S) distribution contains two species, a slow component with invariant *S*_*20,w*_ of 2.31 ± 0.04 (A273V) and 2.30 ± 0.05 (I269F) and fast-moving species with a concentration-dependent *S*_*20,w*_ (2.87–3.18) that constitutes a fraction of about half of the total mass at the highest concentrations measured. Analysis of the molecular weights derived from these data identifies the 2.3 S species as the p18m Dimer-1 and, similar to WT p18m, both mutants retain the capacity to further associate into the higher-order species represented by the fast component.

The affinity of self-association interactions for the interface mutants was measured using multispeed SE-AUC. Sedimentation equilibrium distributions for p18m-A273V and p18m-I269F were recorded at three speeds and varying protein concentrations (Supplementary Table 3 and Supplementary Fig. 6b, c) and the data fitted globally to a monomer-dimer-tetramer model. For both mutants, the best fit was with a tightly associating monomer-dimer equilibrium (*K*_*D*_^*(1-2)*^ = 0.34 µM, A273V) and (*K*_*D*_^*(1-2)*^ = 0.74 µM, I269F) together with a weakly associating dimer-tetramer equilibrium (*K*_*D*_^*(2-4)*^ = 51.8 µM, A273V) and (*K*_*D*_^*(2-4)*^ = 45.3 µM, I269F). These values are largely comparable with that observed for WT p18m, confirming that A273V and I269F maintain the capacity for self-association.

### p18m dimer stability

CD spectroscopy was used to analyse the secondary structure content and examine protein stability of p18m and the A273V and I269F mutants. Far UV CD spectra, 190–260 nm, of p18m, p18m-A273V, and p18m-I269F were recorded at 10 °C (Supplementary Fig. 6d). The spectra essentially overlay and contain a large negative differential molar extinction (*Δε*) at 222 nm, representative of a predominantly α-helical protein and consistent with the crystal structure. In addition, these spectra demonstrate that introduction of the mutations does not result in large rearrangements or loss of protein secondary structure. The stability of p18m, A273V, and I269F mutant dimers was examined by thermal denaturation monitored by far UV CD (Fig. 5d). For WT and mutants, the melting profiles were biphasic with transition midpoints (*T*_*m*_). Irreversibility of the thermal denaturation precluded a Van’t Hoff analysis to detect temperature-dependent changes in *K*_*D*_. However, analysis of *T*_*m*_ derived from derivative plots gives values of 50.7 ± 0.4 °C for p18m and 45.8 ± 0.2 °C and 45.1 ± 0.3 °C for A273V and I269F mutants respectively, showing that these amino acid substitutions modestly reduce protein stability. Nevertheless, the data support the notion that A273V and I269F mutations at the p18m dimer interface are largely tolerated, in accord with the sedimentation data that showed only small differences in both *K*_*D*_^*(1-2)*^ and *K*_*D*_^*(2-4)*^. Moreover, they support the in vivo data demonstrating that when A273V or I269F mutations are introduced into Ty1-Gag, transposition is largely unaffected and when introduced into p18m_AUG1_, they still confer CNC on Ty1.

### Crystal structure of p18m-A273V

To further examine the effects of the A273V mutation, we determined the crystal structure of p18m_AUG2_-A273V (Supplementary Table 2). The protein crystallised in the same spacegroup as p18m with the same three copies arranged as two dimers in the ASU, but now with additional electron density for the A to V substitution on α1 (Fig. 5e). Superposition of the three monomers shows the backbone conformation is near identical (RMSD = 0.16 ± 0.04 Å over 78 ± 3.6 Cα) (Fig. 5f) as was also observed with WT p18m (Fig. 2b). In addition, the Dimer-1 interface contains the same set of apolar and H-bond interactions as in the WT structure. The only difference is the alanine to valine substitution located at the interface centre (Fig. 5g, h). In p18m, the β-methyl groups of A273 contribute to the continuous apolar network that stabilises the dimer and packs across the interface at a favourable Van der Waals spacing of 3.6 Å. In p18m-A273V, it is apparent that the γ-methyl groups of V273 now also form part of the continuous apolar network. However, in order to accommodate the additional methyl groups, there is a small displacement in the backbone position at the C-terminus of α1 in both monomers. As a result of this shift, the V273 γ-methyl groups also pack across the dimer interface and maintain the same favourable 3.6 Å Van der Waals spacing as the β-methyl groups of A273 in the p18m structure. Analysis of the energetic contribution from A273 or V273 to the dimer interface using PDBePISA^52^ also suggests that both the A273–A273 and V273–V273 interactions are favourable and that V273–V273 packing actually contributes more than the A273–A273 packing to the free energy of the overall interaction (Fig. 5g).

### VLP association of p18m interface mutants

**Fig. 6:**
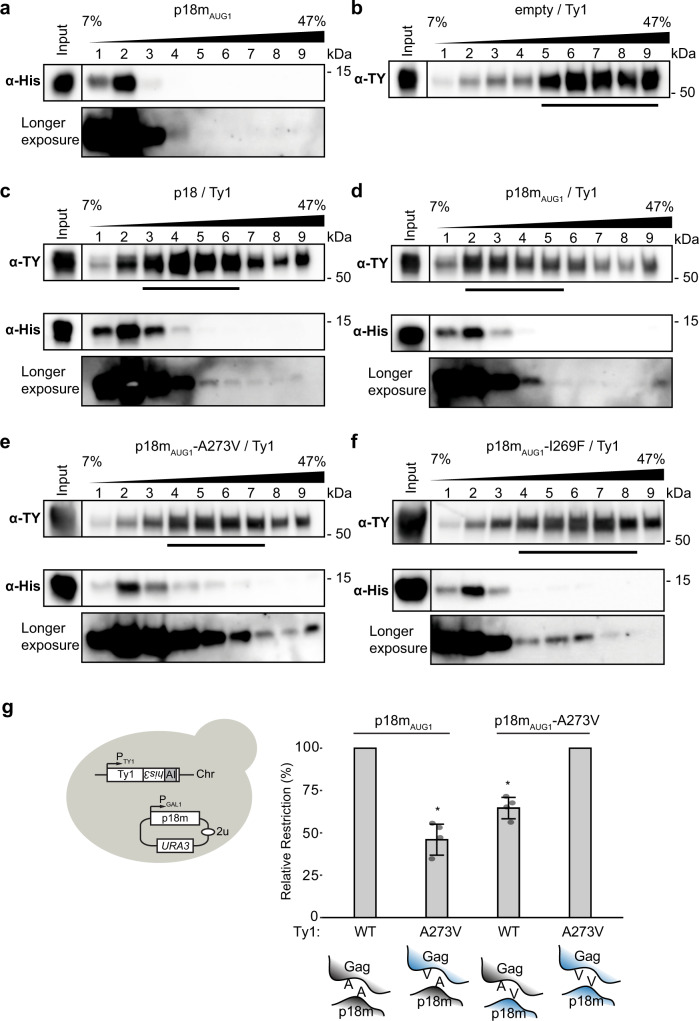
p18m-Gag interaction is critical for restriction activity. **a–f** Protein extracts from galactose-induced yeast cells (Input) were fractionated over a 7–47% continuous sucrose gradient and immunoblotted. The bars below anti-TY blots denote peak Gag fractions containing more than 1/9 of the Gag signal across the gradient, as determined by densitometric analysis. A representative image of at least 3 replicates is shown. Strains used: DG4292 (**a**), DG3739 (**b**), DG4162 (**c**), DG4147 (**d**), DG4165 (**e**), DG4340 (**f**). Migration of molecular weight standards is shown alongside the immunoblots. Images of the whole gel immunoblots are provided in the Source Data file. **g** (Left) Schematic illustrating endogenously expressed, chromosomal Ty1 and galactose-inducible, plasmid-borne p18m_AUG1_. Ty1 is tagged with the *his3-AI* retromobility indicator gene; histidine prototrophy requires retromobility. (Right) Relative restriction by p18m_AUG1_ and p18m_AUG1_-A273V of Ty1 and Ty1-A273V for homotypic and heterotypic pairings. The relative restriction is calculated as the percentage of fold-restriction by the homotypic pairing. Quantitative mobility data is mean from four replicates of galactose-induced cells. Each bar represents the mean of the four independent measurements displayed as points. The error bar centre represents the mean of the four measurements and the error bar extent ± the standard deviation. Significance is calculated from a two-sided Student’s *t*-test compared with homotypic relative restriction (*p < 0.05, exact *p*-values provided in Supplementary Table 1). The cartoons below illustrate homotypic or heterotypic p18m-Gag interactions. See also Supplementary Table 1 and Supplementary Fig. 7. Source data is provided in the Source Data file.

As full-length p18/p22 co-sediments with Ty1 VLPs^25,28^ when inducibly co-expressed, we analysed the sedimentation of p18m_AUG1_ from protein extracts of yeast expressing p18m_AUG1_ and Ty1 in 7–47% continuous sucrose gradients (Fig. 6). In the absence of Ty1 expression, p18m_AUG1_ accumulated in less dense fractions at the top of the gradient (Fig. 6a). In the absence of the restriction factor, Ty1 VLPs accumulated in more dense sucrose fractions towards the bottom of the gradient, with peak fractions indicated by a bar (Fig. 6b). When Ty1 was co-expressed with full-length p18 or p18m_AUG1_, a minor fraction of the restriction factor appeared in higher density fractions (Fig, 6c, d), although the highest concentrations of p18 and p18m_AUG1_ remained at the top of the gradient. This is similar to results obtained with p22^25,28^. p18m_AUG1_-A273V and I269F also fractionated with VLPs (Fig. 6e, f), consistent with our data confirming their restriction activity. As previously reported^25,28^, the most striking effect we observe is a redistribution in the Gag fractionation pattern in the presence of restriction factor, with a shift of Ty1 Gag towards the top of the gradient and an overall broadening of peak fractions (Fig. 6c–f). This redistribution is consistent with p18m_AUG1_ interfering with Gag oligomers required to assemble complete VLPs, as the aberrant Gag-complexes do not sediment as far into the gradient. However, the binding dynamics between p18m_AUG1_ and VLP assembly intermediates are likely complex and influenced by the relative amounts of the restriction factor and Gag, and the fact that p18m and Ty1 are co-expressed in the absence of pre-existing Ty1 gene products.

### p18m restriction of a CNC-resistant Ty1 element

Since p18m_AUG1_ fractionates with VLPs, is identical to the Gag CA-CTD, and the CNC^R^ mutant Gag-A273V^28^ is within the critical p18m Dimer-1 interface, p18m CNC might be mediated by a p18m-Gag interaction within this region. However, the relative level of Gag and p22/p18 and timing of expression influence restriction, as evidenced by loss of CNC-resistance when wild type p22 is inducibly co-expressed at a higher level than a Ty1 Gag-CNC^R^ mutant^28,53^. Here, we showed that p18m_AUG1_-A273V restricted Ty1 mobility as well as wild-type p18m_AUG1_ (Fig. 4d–f), raising the possibility that inducible co-expression of Ty1 and p18m_AUG1_-A273V may mask the effect of a heterotypic interaction. In support of this notion, uncoupling Ty1 and restriction factor expression has been utilized to identify a Gag/p22 ratio optimal for isolating CNC^R^ mutants such as Gag-A273V^28^.

Therefore, we uncoupled Ty1 and p18m_AUG1_ expression to explore protein interactions genetically. Isogenic strains containing an endogenously expressed chromosomal insertion of a WT or A273V Ty1*his3-AI* element were analysed for Ty1 mobility following inducible expression of p18m_AUG1_, p18m_AUG1_-A273V or empty vector. Restriction of WT and A273V Ty1 elements by p18m_AUG1_-WT and A273V were compared in a pairwise fashion, normalized to Ty1 mobilities in the absence of the restriction factor, and strains were verified for comparable levels of Gag-A273V and p18m_AUG1_-A273V relative to WT (Fig. 6g, Supplementary Fig. 7 and Supplementary Table 1). WT-WT or A273V-A273V restricted significantly better than heterotypic pairings of p18m_AUG1_ and Ty1. These results support a model of p18m_AUG1_ restriction in which Dimer-1 residues interact with the corresponding residues in Gag and interfere with proper Gag function. The heterotypic pairing of Gag-A273V with p18m_AUG1_-A273 may also contribute to CNC-resistance.

## Discussion

Genetic dissection of Ty1 initially showed that a C-terminal domain in Gag (UBN2) is contained within the retrotransposon restriction factor p22/p18^28^ (Fig. 1a). Our crystallographic and biophysical analyses of bacterially expressed p18m derived from this region greatly extend these genetic studies by revealing that UBN2-p22 is highly related to the Gag CA-CTD of several retroelements or exapted Gag proteins^54–56^. DALI comparisons reveal weaker but significant matches to CA proteins of infectious retroviruses such as MLV and HIV-1 as well as the human endogenous retrovirus HERV-K (Supplementary Fig. 1). Our findings expand the view that the CA gene of LTR retrotransposons share a common evolutionary origin^41,42,57^.

Biophysical studies of p18m reveal a stable obligate dimer with a tendency to form higher-order structures, and crystal studies indicate the presence of two possible dimer interfaces in p18m (Fig. 2). Dimer-1 involves a largely hydrophobic interaction between the exposed surfaces of α1 and α3 on two opposing monomers whereas the Dimer-2 interaction is through the side chains of residues exposed on the exterior of α4 and α5. The importance of Dimer-1 for p18m integrity and function is evident from mutational analyses in bacteria and yeast as introduction of changes likely disruptive to Dimer-1 results in loss of protein integrity and only conservative changes are tolerated (Supplementary Table 4). The core Dimer-1 interface residues I269 and A273 are generally more sensitive to disruption both in the context of Gag and p18m. However, whilst Gag is more tolerant to polar substitutions at residues V270 and L312, just peripheral to the core, the L312S mutation is not tolerated in the context of p18m.

NNK mutagenesis revealed exceptional substitutions in the Dimer-1 core interface as p18m_AUG1_ tolerates A273S and A273C. Bacterially expressed p18m_AUG2_-A273C still forms strong dimers under reducing conditions when assessed by MALLS (Supplementary Fig. S8a) and mass spectrometry analysis demonstrates there are no covalently linked chains (Supplementary Fig. S8b). While the rules governing the Dimer-1 interface remain incomplete, our data imply that Gag likely has more binding interfaces to stabilize the protein than does the p18m fragment, which appears to form obligate dimers relying on the hydrophobic Dimer-1 interface to retain restriction factor activity.

An essential step in the replication of retroviruses and LTR retrotransposons involves the assembly of Gag into a shell surrounding their RNA genomes. These fullerene structures are made up of arrays of hexameric and pentameric Gag or CA. A number of studies employing cryo-electron tomographic and single-particle analysis of native viral particles or in vitro assemblies have been performed to study these shell structures^32–37^ and to characterise the interactions involving the NTDs and CTDs of CA. These include the NTD-NTD interactions that build the hexamer or pentamer, the CTD-CTD interactions that link the neighbouring capsomeres and NTD-CTD interactions that stabilize the overall structure. Importantly, the architecture of the CTD-CTD interaction is highly conserved. Inspection of the p18m Dimer-1 interface from Ty1 reveals that it is highly related to dimer interfaces in Ty3^41^ and the dARC proteins from *Drosophila*^42,43^, and more distantly to retroviral structures (Supplementary Fig. 2). Given these CA-CTD dimer structures all contain the same hydrophobic core and surrounding salt bridges, this implies a similar role for Dimer-1 in Ty1 VLP assembly.

The importance of the Dimer-2 interface for p18m structure and Gag function is revealed by biophysical and genetic analyses, and molecular modelling. The p18m-F323S substitution within α4 suppresses higher-order oligomers but does not affect Dimer-1 interaction (Fig. 3d), raising the possibility of another function for Dimer-2. Previous genetic analyses show that an in-frame codon insertion at Gag-I341 affects transposition, amino acid substitution Gag-I343K within α5 alters VLP assembly^58–60^, and Gag-V336I within α5 confers weak CNC-resistance^28^. Here, we characterise F323 substitutions of Dimer-2 in the context of p18m_AUG1_ and Gag. Remarkably, a separation of function phenotype is observed for F323S and F323D. Neither substitution affects restriction (Fig. 3e, f), but both greatly decrease Ty1 mobility when placed in *GAG* (Fig. 3g, h). The data suggest that the Dimer-2 interface may maintain the Ty1 Gag particle 3-fold axis (Supplementary Figure 4) that is required for normal VLP assembly and transposition but is not required for p18m_AUG1_ restriction.

**Fig. 7:**
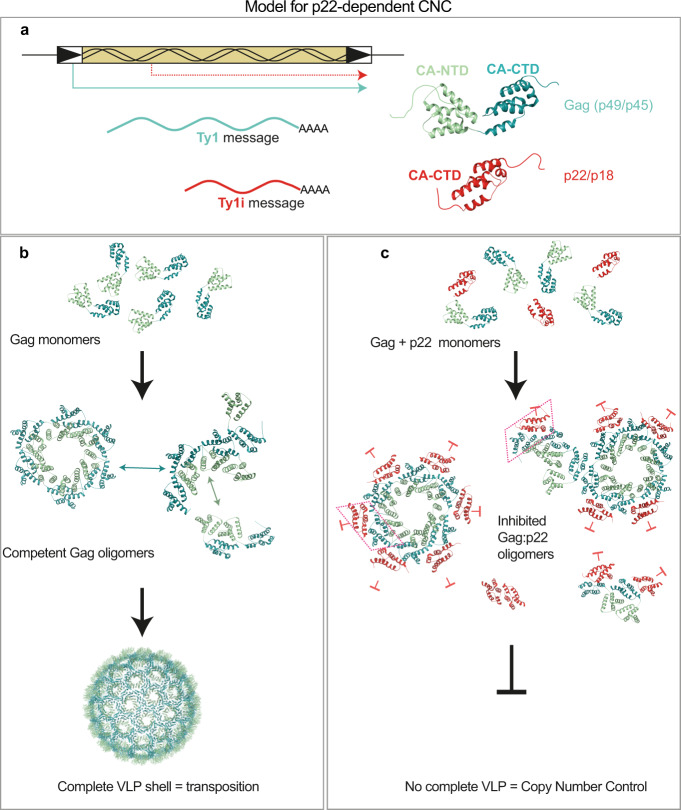
Model for p22/p18-dependent CNC. **a** Transcription of Ty1 results in either a full-length Ty1 transcript (teal), or a short, inhibitory transcript Ty1i (red). The long transcript encodes full-length Ty1 Gag and Pol proteins, shown in green/cyan cartoon. The shorter Ty1i transcript encodes p22/p18, a C-terminal portion of Gag equivalent to a CA-protein CTD, lacking an NTD (red cartoon), as well as Pol proteins, which are not required for restriction^25^. **b** Full-length Gag monomers assemble into oligomers through NTD-NTD, and CTD-CTD interactions to construct an icosahedral shell, constituting the VLP, which is required for transposition. **c** In the presence of p22/p18, Gag monomers are also able to oligomerise with p22/p18 through CTD-CTD interactions. Two examples of a Gag-p22/p18 hetero-interaction through the CTD interface are highlighted by the trapezoidal boxes. While Gag proteins may be able to homo-oligomerise through their NTDs, Gag oligomers that associate with p22/p18 become assembly dead ends resulting in partially assembled particles that cannot support transposition.

Given that p18m is identical to the CA-CTD of Ty1 Gag, we propose a structural model for CNC, where the insertion of p22/p18 into the VLP lattice during assembly produces non-productive or dead-end interactions (Fig. 7). In the normal course of VLP formation, Ty1 Gag assembles into complete VLPs through NTD-NTD, NTD-CTD, and CTD-CTD interactions that are all required to form a closed intact shell. If p18m is introduced via a homotypic interaction with the CTD of Gag, as suggested by pairwise interactions of p18m and Gag (Fig. 6g), normal assembly is unable to further propagate because it lacks an NTD. This will result in partial VLP and/or incomplete lattice structures. The dispersion of VLP assemblies following sucrose gradient sedimentation also supports this model (Fig. 6a–f**)**. Since the state of capsid assembly is essential for productive reverse transcription in both the spuma- and ortho-retroviruses^61–63^, the specific architecture of Ty1 VLPs should also affect critical steps in the Ty1 replication cycle. Indeed, several defects in the process of Ty1 retrotransposition are detected during CNC^24,53^. Reverse transcription intermediates and full-length cDNA are not detected, PR cleaves Pol proteins aberrantly, mature IN fails to accumulate, and Ty1 RNA is more sensitive to ribonuclease treatment^25,27,28,64,65^. We hypothesize that some limited incorporation of p22 is tolerated, allowing PR-mediated conversion of p22 to p18. However, once a threshold level of p22/p18 is reached, it would become impossible to build the stable VLP shells required for protein maturation, reverse transcription, and integration due to Gag oligomers becoming poisoned by the incorporation of p18.

Analysis of the Gag-A273V CNC^R^ mutation also supports the idea that a homotypic interaction at the Dimer-1 interface enhances VLP formation. In the context of p18m, A273V subtly alters the p18m Dimer-1 interface (Fig. 5) and in the context of Gag, A273V does not affect transposition (Fig. 4). However, Gag-A273V confers resistance to p22 and attenuates PR-mediated processing of p22–p18^28^, suggesting that structural changes affect access to PR and CNC^R^ in vivo. Here, we provide evidence that heterotypic pairings of A273 and A273V on p18m and Gag promote CNC^R^ when compared with homotypic pairings (Fig. 6g). Therefore, CNC^R^ may result from the reduced association at the Dimer-1 interface between heterotypic p18m and Gag-A273V during VLP assembly.

Data from the two yeast co-expression systems utilized here support the idea that p18m interferes at an early VLP assembly step and raises additional questions concerning the resistance of preformed Gag-complexes to p18m. In a Ty1-less strain background, ectopic overexpression of p18m and Ty1 from galactose-inducible promoters results in potent restriction (Figs. 1 and 4 and Supplementary Table 1)^25,28^. However, the same galactose-induced p18m against endogenously expressed Ty1 results in much lower restriction (Fig. 6G and Supplementary Table 1). Constitutive native expression of a chromosomal Ty1 element may allow threshold steps in VLP assembly to begin prior to induction of p18m. The strong resistance that early expression confers on Ty1, independent of any mutations, implies an important but incompletely defined kinetic component to CNC, and suggests p22 acts at an early VLP assembly step, consistent with our model.

Importantly, our model explains the negative feedback loop proposed for CNC^24–26^. As the Ty1 copy number increases, the expression of p22, and therefore sequestration of Gag, also increases. At a certain Ty1 copy number, the p22 threshold level is reached, thus stabilising the number of genomic elements, and preventing runaway transposition from compromising the host genome. In the reference strain S288C, this appears to be ~32 Ty1 elements. Nevertheless, differing affinities of the CTD-CTD interface, expression of p22, or horizontal transfer of novel *GAG* sequences from other species might modulate this threshold level in related elements or different strains, resulting in differing genomic copy numbers to reach the threshold level of p22^6^.

Ty1 CNC mediated by p22 is related to other Gag-like restriction factors active against retroviruses. Precedence for restriction through the interference of assembly comes from endogenous HERV-K inhibition of HIV-1 particle assembly^66,67^. In addition, ovine restriction factors derived from endogenous Jaagsiekte retroviruses are altered Gag proteins that block viral Gag trafficking during a late stage of oncogenic Jaagsiekte retroviral infection^68,69^. Gag-like restriction factors also act at the post-entry phase of retroviral infection. Fv1 is an exapted restriction factor encoded by an endogenous retroelement *GAG* gene that has been active in the *Muroidea* superfamily of mammals for at least 45 million years^70,71^. Fv1 alleles selectively restrict MLV infection at a step between reverse transcription and integration through interactions with the Gag core^72^. Comparative structural analyses between Fv1 and HIV restriction factors Trim5α and TrimCypA reveal how the extended antiparallel organization of the dimeric restriction factor enhances their affinity for a preformed HIV lattice^73^.

Our work raises the possibility that CA-CTD, CA-NTD domains, or perhaps additional coding segments derived from *GAG* or *POL* exist as exapted restriction factors active against infectious or endogenous retroelements. Genome sequence comparisons reveal *S. cerevisiae* strains that contain truncated Ty sequences with coding potential at high allele frequency^6,74,75^. There is also a growing body of evidence for the exaptation of endogenous retroviral gene segments in vertebrates^54,76,77^, with many of these involving *GAG*.

## Methods

### Yeast strains and plasmids

Strains and plasmids are listed in Supplementary Table 5 and Supplementary Table 6, respectively. Standard yeast genetic and microbiological techniques were used in this work^78^. All Ty1 nucleotide and amino acid information correspond to the Ty1H3 sequence (GenBank M18706.1)^79^. p*GAL*-Yes2 (pBDG1293, Invitrogen cat. no. V825-20) derived plasmids were generated by cloning custom commercial gene fragments (Integrated DNA Technologies and Twist Bioscience) using XhoI and EcoRI with NEBuilder HiFi DNA Assembly Master Mix (New England Biosciences cat. no. E2621). All plasmids generated were verified by DNA sequencing.

### Yeast Media

For galactose induction in liquid media, starter cultures were grown overnight at 30 °C in synthetic media containing 2% raffinose, diluted 1:20 into media containing 2% galactose, and grown at 22 °C.

### Ty1*his3*-AI mobility assays

Ty1 retromobility events were detected using the *his3-AI* retromobility indicator gene^44^ by qualitative and quantitative assays^25^. Qualitative assays were printed from glucose plates onto galactose plates, grown for 48 h at 22 °C, then printed to glucose plates lacking histidine and grown at 30 °C. Quantitative retromobility frequencies were determined from quadruplicate galactose inductions diluted in water, plated on synthetic dropout media, and colonies counted. All experiments were galactose-induced for 48 h at 22 °C, except for strains DG4296-98 and DG4279-81 which were galactose-induced for 24 h. Data represent at least four independent galactose inductions; *p*-values were calculated by two-sided Student’s *t*-test. Complete data, including standard deviations and *p-*values, are listed in Supplementary Table 1.

### Immunoblotting

Immunoblotting of total protein from galactose-induced yeast prepared by trichloroacetic acid (TCA) precipitation was performed using standard techniques^25^. Cells were broken by vortexing in the presence of glass beads in 20% TCA and washed in 5% TCA. Proteins were separated on 15% (for detecting p18 constructs), 10% (for detecting Pgk1), or 8% (for detecting Gag) SDS-PAGE gels. PVDF membranes were immunoblotted with antibodies at the following dilutions in 2.5% milk-TBST: polyclonal rabbit p18 antisera (1:5000)^25^, monoclonal rabbit hexa-histidine antibody clone RM146 (ThermoFisher cat. no. MA5-33032) (1:1000), mouse monoclonal anti-TY tag antibody clone BB2 (1:5000)^80^ or mouse monoclonal anti-Pgk1 antibody clone 22C5D8 (Invitrogen cat. no. 459250) (1:1000). Immune complexes were detected with WesternBright enhanced chemiluminescence (ECL) detection reagent (Advansta cat. no. K-12049-D50). All imaging was done using a ChemiDoc MP (Bio-Rad). Precision Plus Kaleidoscope protein standards (Bio-Rad cat. no. 1610395) were used to estimate molecular weights. Total protein was detected by running samples on a 10% TGX Stain-Free^TM^ FastCast^TM^ Acrylamide gel (Bio-Rad cat. no. 1610173) and gel-imaging after 45 s of activation. Protein quantification with total protein normalization was performed using Image Lab (Bio-Rad).

### Protein expression and purification

The DNA sequence for *S. cerevisiae* Ty1A (Uniprot P08405), codon-optimised for expression in *E. coli*, was synthesised by GeneArt. Sequences corresponding to residues M249-N355 (p18m_AUG1_) and M259-N355 (p18m_AUG2_) were amplified by PCR and the products were inserted into a pET22b expression vector (Novagen) between the NdeI and XhoI restriction sites in order to produce C-terminal fusion proteins containing the hexa-histidine tag PLEHHHHHH. Mutations were introduced into these parent constructs using the Quikchange II XL site-directed mutagenesis kit (Agilent) following the manufacturer’s instructions. The codon optimised p18m DNA sequence and primer sequences for PCR and mutagenesis are provided in Supplementary Table 7.

p18m proteins were expressed in the *E. coli* strain BL21 (DE3) grown in LB-broth by induction of log-phase cultures with 1 mM IPTG, followed by incubation overnight at 20 °C with shaking. Cells were pelleted and resuspended in 50 mM Tris-HCl, 150 mM NaCl, 10 mM Imidazole, 5 mM MgCl_2_, 1 mM DTT, pH 9.0, supplemented with 1 mg mL^−1^ lysozyme (Sigma-Aldrich), 10 µg mL^−1^ DNase I (Sigma-Aldrich), and 1 Protease Inhibitor cocktail tablet (EDTA free, Pierce) per 40 mL of buffer. Cells were lysed using an EmulsiFlex-C5 homogeniser (Avestin) and His-tagged protein captured from the clarified lysate using immobilised metal ion affinity on a 5 mL Ni^2 + ^-NTA Superflow column (Qiagen).

For crystallographic analysis of p18m_AUG1_ and p18m_AUG2_-A273V, Ni^2 + ^-NTA bound proteins were eluted with 50 mM Tris-HCl, 150 mM NaCl, 250–300 mM Imidazole pH 9.0. Carboxypeptidase A (Sigma C9268) was added at 1:100 (w:w) ratio and the resulting mixture was incubated overnight at 4 °C to digest the C-terminal his-tag. The Carboxypeptidase A was then inactivated by the addition of 2 mM Tris (2-carboxyethyl) phosphine (TCEP) and proteins further purified by gel filtration chromatography on a Superdex^TM^ 75 (26/60) column equilibrated in 20 mM Tris-HCl, 150 mM NaCl, 1 mM TCEP pH 9.0.

For p18m_AUG2_, the protein was transferred to Acetate Buffer (50 mM Sodium Acetate, 300 mM NaCl, 1 mM TCEP pH 5.0). The, Ni^2 + ^-NTA eluent was first diluted 1:4 with 2 × Acetate Buffer, the pH adjusted to 5.0 with HCl, and then the protein dialysed exhaustively against 2 L of Acetate Buffer overnight (SnakeSkin dialysis tubing, 10 kDa MWCO, ThermoFisher). p18m_AUG2_ was then further purified by gel filtration chromatography on a Superdex^TM^ 75 (26/60) column equilibrated in Acetate Buffer. Seleno-methionine p18m_AUG2_ was produced using an identical procedure, except *E. coli* B834 (DE3) cells, grown in seleno-methionine Medium (Molecular Dimensions, Newmarket, UK), were used to express the protein.

Electrospray-ionisation mass spectrometry (ESI-MS) was used to determine protein molecular masses of WT and mutants, ascertain the degree of seleno-methionine incorporation, and confirm His-tag removal where appropriate. Usually, complete digestion left a C-terminal PLEH remnant. Purified proteins were concentrated by centrifugal ultrafiltration (Vivaspin, MWCO 10 kDa), then snap-frozen and stored at −80 °C. Protein concentrations were determined by UV absorbance spectroscopy using a calculated extinction coefficient at 280 nm.

### Protein crystallisation

p18m proteins were crystallised using sitting drop vapour diffusion at 18 °C, using Swissci MRC 2-drop trays (Molecular Dimensions) with drops set using a Mosquito robot with humidity chamber (TTP Labtech).

Initial trials using native p18m_AUG2_ produced only poorly diffracting fibrous needles. However, crystals of Se-Met p18m_AUG2_ were obtained using 13.6 mg mL^−1^ protein in 50 mM Sodium Acetate pH 5.0, 300 mM NaCl 1 mM TCEP and mother liquor containing glycerol and PEG 4 K. Optimisation of these conditions including microseeding (Seed Bead kit, Hampton) produced the best crystal in a condition containing a mixture of 260 nL protein (13.6 mg mL^−1^), 120 nL precipitants (27.9% Glycerol, 17.7% PEG 4 K 0.1 M HEPES pH 7.5), 20 nL seed solution (seeds produced in a solution of 100% mother liquor of 20% Glycerol, 31% PEG 4 K 0.1 M HEPES pH 7.5). The large majority of drops with this condition produced crystals that were over-nucleated thin needles or spherulites. However, in one drop a hexagonal crystal appeared after ~8 days and reached a maximal size of 80 × 80 × 250 µm after ~21 days before it was harvested into liquid nitrogen using a lithographic loop (MiteGen) and mother liquor as cryoprotectant.

Well-diffracting crystals of p18m_AUG1_ were obtained with 6.25 mg mL^−1^ protein in 20 mM Tris-HCl pH 8.5, 150 mM NaCl, 1 mM TCEP using 200 nL protein solution, and 200 nL of mother liquor with pH ranging from 7.5–9.0 and containing between 1.125–1.250 M Li_2_SO_4_. The best crystal was a hexagonal prism ~160 × 160× 160 µm and was harvested into liquid nitrogen from a drop containing 1.16 M Li_2_SO_4_, 0.1 M Tris-HCl pH 9.0 using a cryoprotectant of 1 M Li_2_SO_4_, 0.1 M Tris-HCl, 2 M Sodium Malonate, pH 7.5. p18m-A273V crystals were obtained under similar conditions, although the quality of the crystals was consistently worse. The best diffracting crystal grew in a 400 nL drop containing 200 nL protein (30 mg mL^−1^) and 200 nL mother liquor (1.16 M Li_2_SO_4_, 0.1 M MES pH 6.59). Crystals were harvested into liquid nitrogen using the same cryoprotectant as for p18m_AUG1_.

### Data collection and structure determination

Data from Seleno-methionine crystals of p18m_AUG2_ were collected at the Diamond Light Source (DLS) tuneable beamline, I04. Grid-scanning was required to ensure that only a well-diffracting portion of the crystal was exposed to the beam and a fluorescence excitation scan was recorded to determine the best wavelengths for the collection of multi-wavelength anomalous dispersion (MAD) data. A high-redundancy peak-wavelength dataset was collected using an inverse-beam strategy to maximise the preservation of anomalous signals. Inverse-beam dataset halves were processed using the Xia2 pipeline^81^, using DIALS^82^ and the integrals were scaled on rotation axis before merging using AIMLESS^83^, yielding a single dataset with very strong anomalous differences to 3.76 Å resolution. Subsequent datasets were collected at a high-energy remote and the inflection wavelength, with lower exposure and redundancy to reduce radiation damage. These datasets were processed using the xia2 pipeline using DIALS and AIMLESS, and also contained significant anomalous differences (Supplementary Table 2).

MAD Phasing was undertaken using the SHELX suite of programs^84^. Solutions for phases in P6_5_22 gave interpretable experimental density maps. Further solvent flattening using SHELXE resulted in maps that enabled the manual building of a complete model aided by the helix-finding and baton building tools of COOT^85^. Initial register assignment was possible due to the density of large sidechains and the positions of Seleno-methionine residues. The model was refined in PHENIX^86^ using the high-resolution peak-dataset and separated Bijvoet pairs to account for the strong anomalous signal in the data.

Diffraction data from p18m_AUG1_ and p18m_AUG2_-A273V crystals were collected at DLS beamlines I03 and I04 respectively. Data were processed using the Xia2 pipeline using DIALS and AIMLESS. The structures were solved by molecular replacement in PHASER^87^ implemented in the CCP4 interface^88^, using the p18m_AUG2_ monomer as a search model.

All datasets had the same spacegroup and cell dimensions, so a consistent R_free_ test set was enforced across all refinement datasets in PHENIX and REFMAC5^89^. TLS groups, were determined using TLSMD^90^ and included in the final rounds of refinement when models were near complete. Throughout refinement, the model geometry was monitored and assessed using Molprobity^91^ and PDB-REDO^92^. Details of data collection, phasing, and structure refinement statistics are presented in Supplementary Table 2.

### Structure analysis and alignments

Molecular interfaces were analysed using the EBI protein structure interface analysis service PDBePISA (https://www.ebi.ac.uk/pdbe/pisa). The Surface hydrophobicity/hydrophilicity distribution was calculated using the Pymol script (https://pymolwiki.org/index.php/Color_h). The DALI comparison server (http://ekhidna2.biocenter.helsinki.fi/dali) was used to search for and align structural homologues from the PDB. For sequence alignments and conservation analysis, *Saccharomyces* spp genomes obtained from SGD (https://www.yeastgenome.org/) were searched with tBLASTn for intact Ty1-type capsid ORFs using e-value settings determined to exclude Ty2 elements and other transposons (‘-evalue 1e-90’). Obtained sequences were translated, filtered to retain only those with ≥90% coverage of capsid, which was oriented and aligned with MAFFT (‘linsi –adjustdirection –reorder’) v7.453^93^ and used to form a maximum-likelihood tree with FastTree v2.1.11^94^. To increase the accuracy and stringency of downstream analyses, where unknown amino acid (X residue) positions could be unequivocally inferred based on the position of the sequence within the tree, determined residues were incorporated into the sequences. In total, 125 sequences were retained and used to calculate conservation scores, each corresponding to a site’s evolutionary rate, using ConSurf^48^, these scores being used to colour the structure according to conservation. A reduced representation of the 125-sequence alignment was obtained by the selection of exemplar sequences based on the frequency of their clades within the maximum-likelihood tree. The residue colouring in the alignment is according to Clustal W^95^.

### SEC-MALLS

Size exclusion chromatography coupled multi-angle laser light scattering (SEC-MALLS) was used to determine the molar mass distribution of p18m and p18m interface mutants. Samples ranging from 100–400 µM were applied in a volume of 100 µL to a Superdex^TM^ INCREASE 200 10/300 GL column equilibrated in Acetate Buffer at a flow rate of 1.0 mL min^−1^. The scattered light intensity and the protein concentration of the column eluate were recorded using a DAWN-HELEOS laser photometer and OPTILAB-rEX differential refractometer respectively. The weight-averaged molar mass of material contained in chromatographic peaks was determined from the combined data from both detectors using the ASTRA software version 7.3.2.19 (Wyatt Technology Corp., Santa Barbara, CA, USA).

### Analytical ultracentrifugation

Sedimentation velocity experiments were performed in a Beckman Optima Xl-I analytical ultracentrifuge using conventional aluminum double sector centrepieces and sapphire windows. Solvent density and the protein partial specific volumes were determined as described^96^. Prior to centrifugation, p18m and p18m interface-mutant samples were prepared by exhaustive dialysis against the buffer blank solution (Acetate Buffer). Samples (420 µL) and buffer blanks (426 µL) were loaded into the cells and centrifugation was performed at 50,000 rpm (182,000 × *g*) and 293 K in an An50-Ti rotor. Interference data were acquired at time intervals of 180 s at varying sample concentrations (30–181 µM) using the ProteomeLab 6.04 software. Data recorded from moving boundaries were analysed in terms of the continuous sedimentation coefficient distribution function C(S) using the program SEDFIT^97^.

Sedimentation equilibrium experiments were performed in a Beckman Optima XL-I analytical ultracentrifuge using aluminum double sector centrepieces in an An-50 Ti rotor. Prior to centrifugation, p18m and p18m interface-mutant samples were dialyzed exhaustively against the buffer blank (Acetate Buffer). Samples (150 µL) and buffer blanks (160 µL) were loaded into the cells and after centrifugation for 30 h, interference data were collected at 2 h intervals until no further change in the profiles was observed. The rotor speed was then increased, and the procedure repeated. Data were collected at three speeds 18,000 rpm (23,587 × *g*), 21,000 rpm (32,105 × *g*) and 26,000 rpm (49,213 × *g*) on samples at different concentrations of p18m, p18m(A273V) and p18m(I269F). The program SEDPHAT^98^ was used to initially determine weight-average molecular masses by nonlinear fitting of individual multispeed equilibrium profiles to a single-species ideal solution model. Inspection of these data revealed that the molecular mass showed significant concentration dependency and gave poor fits to a single species model. Therefore, global fitting of the data to a monomer-dimer-tetramer model incorporating the data from multiple speeds and multiple sample concentrations was applied to extract monomer-dimer (*K*_*D*_^1,2^) and dimer-tetramer (*K*_*D*_^2–4^) equilibrium dissociation constants.

### CD spectroscopy

Far UV CD spectra (260–190 nm) were recorded using a Jasco J-815 spectropolarimeter purged with nitrogen gas and equipped with a Peltier temperature controller. Spectra (25 accumulations) were recorded at 10 °C in 0.1 cm cells at a protein concentration of 150 µg mL^−1^ in 10 mM Na Acetate pH 5.0. All spectra were corrected by subtraction of the appropriate buffer blank.

The melting profile of proteins was monitored by recording the CD at 222 nm whilst heating samples at a constant rate of 1 °C per minute from 10 °C to 80 °C. The melting data were fitted with a spline function and the T_m_ for thermal transitions determined from the maximum of the 1st derivative.

### NNK mutant screen

Gene fragment libraries (Integrated DNA Technologies) containing a randomized NNK codon (N = A/C/T/G, K = G/T) at either Gag-269 or Gag-273 were cloned into a pGTy1*his3-AI*/2μ-*URA3* plasmid [pGTy1mhis3-AI (pBDG598)]^44^. The vector was digested with BbvCI and BstEII, gel purified, and assembled with three overlapping gene fragments using with NEBuilder HiFi DNA Assembly Master Mix (New England Biosciences cat. no. E2621): Ty1_nt.738-989_, Ty1_nt.969-1361_-NNK, Ty1_nt.1341-1828_. The NNK plasmid library was introduced into electrocompetent TOP10 *E. coli* cells (Invitrogen cat. no. C404050). Approximately 1000 bacterial colonies were pooled, the plasmid DNA was extracted by midi-prep (Qiagen cat. no. 12143), then transformed into a Ty1-less yeast strain (DG3582). Yeast transformants were replica plated onto galactose plates to induce Ty1 expression and grown for 2 days at 22 °C, then replica plated onto media lacking histidine, and the level of His^+^ papillation was assessed after 3 days at 30 °C. Mutations were identified by DNA sequencing PCR-amplified regions bracketing the 269 or 273 NNK codon (primer set: 5′-GCCACAATCACAGTTTCCGC-3′ and 5′-TGCTGTGATATCTACTGCAGCC-3′). Select mutations were validated by recovering plasmid from yeast and sub-cloning a PCR product (primer set: 5′-GGTAATACATTTACTGATTCATCCTCAGC-3′ and 5′-CCTGGAAGTGAAATTGTAGG-3′) into HpaI/BstEII digested pBDG1534 (pGTy1his3-AI/*TRP1* Cen) with NEBuilder HiFi DNA Assembly Master Mix (New England Biosciences cat. no. E2621). The plasmid was recovered from yeast after breaking cells by vortexing in the presence of glass beads in TNSTE-PCI (1% Triton X-100, 0.5% SDS, 50 mM NaCl, 5 mM Tris pH 8.0, 0.5 mM EDTA, 50% phenol:chloroform:isoamyl alcohol 25:24:1), then ethanol precipitating the aqueous phase and resuspending in TE. Subcloned mutant plasmids were fully sequenced to verify no secondary mutations accumulated during mutagenesis.

### Sucrose gradient sedimentation

Following 48 h galactose induction, a 100 mL culture was harvested and cells were broken in 15 mM KCl, 10 mM HEPES- KOH, pH 7, 5 mM EDTA containing RNase inhibitor (100 U/mL), and protease inhibitors (16 μg ml^−1^ aprotinin, leupeptin, pepstatin A and 2 mM PMSF) in the presence of glass beads. Cell debris was removed by centrifuging the broken cells at 10,000 × g for 10 min at 4 °C. Approximately five milligrams total protein in 500 μL of buffer was applied to a 7–47% continuous sucrose gradient and centrifuged using an SW41 Ti rotor at 25,000 rpm (77,000 × g) for 3 h at 4 °C. After centrifugation, 9 × 1.2 mL fractions were collected and normalized volumes of input and fractions were immunoblotted with TY-tag antibody to detect Gag and hexa-histidine antibody to detect p18m_AUG1_^28^. Densitometric analysis was performed using Image Lab (Bio-Rad).

### Reporting summary

Further information on research design is available in the Nature Research Reporting Summary linked to this article.

## Supplementary information

Supplementary Information

Reporting summary

## Data Availability

For sequence conservation analysis, *Saccharomyces* spp genomes were obtained from SGD (https://www.yeastgenome.org/). Protein structures used in structural alignments were obtained from the Protein Data Bank (https://www.rcsb.org/). The atomic coordinates and structure factors for p18m_AUG1_, p18m_AUG2_, and p18m_AUG2_-A273V have been deposited in the Protein Data Bank under accession numbers 7NLH [10.2210/pdb7NLH/pdb], 7NLI [10.2210/pdb7NLI/pdb] and 7NLG [10.2210/pdb7NLG/pdb]. The entire p18m sequence alignment is available to download from the Figshare repository (https://crick.figshare.com/articles/dataset/Cottee_Supplementary_datafile1_Ty1p18_alignment_txt/15060366). The Source data and whole blot images underlying Figs. 1d, 1f, 3b-c, 3f-h, 4b-c, 4e-f, 5c-d, 6a-g, and Supplementary Figs 6a–c, and 7 are provided as a Source Data file. All remaining data are contained within the article. Source data are provided with this paper.

## Source data

Source Data
