# Supplementary material for: Structure of a Ty1 restriction factor reveals the molecular basis of transposition copy number control
Source: Nat Commun. 2021 Sep 22;12:5590. doi: 10.1038/s41467-021-25849-0 (PMC8458377; doi:10.1038/s41467-021-25849-0)
Supplement: Supplementary file 1 — Supplementary Information [file 41467_2021_25849_MOESM1_ESM.pdf]

Supplementary information

Structure of a Ty1 restriction factor reveals the molecular basis of transposition copy number control

Matthew A. Cottee, Sean L. Beckwith, Suzanne C. Letham, Sarah J. Kim, George R. Young, Jonathan P. Stoye, David J. Garfinkel and Ian A. Taylor

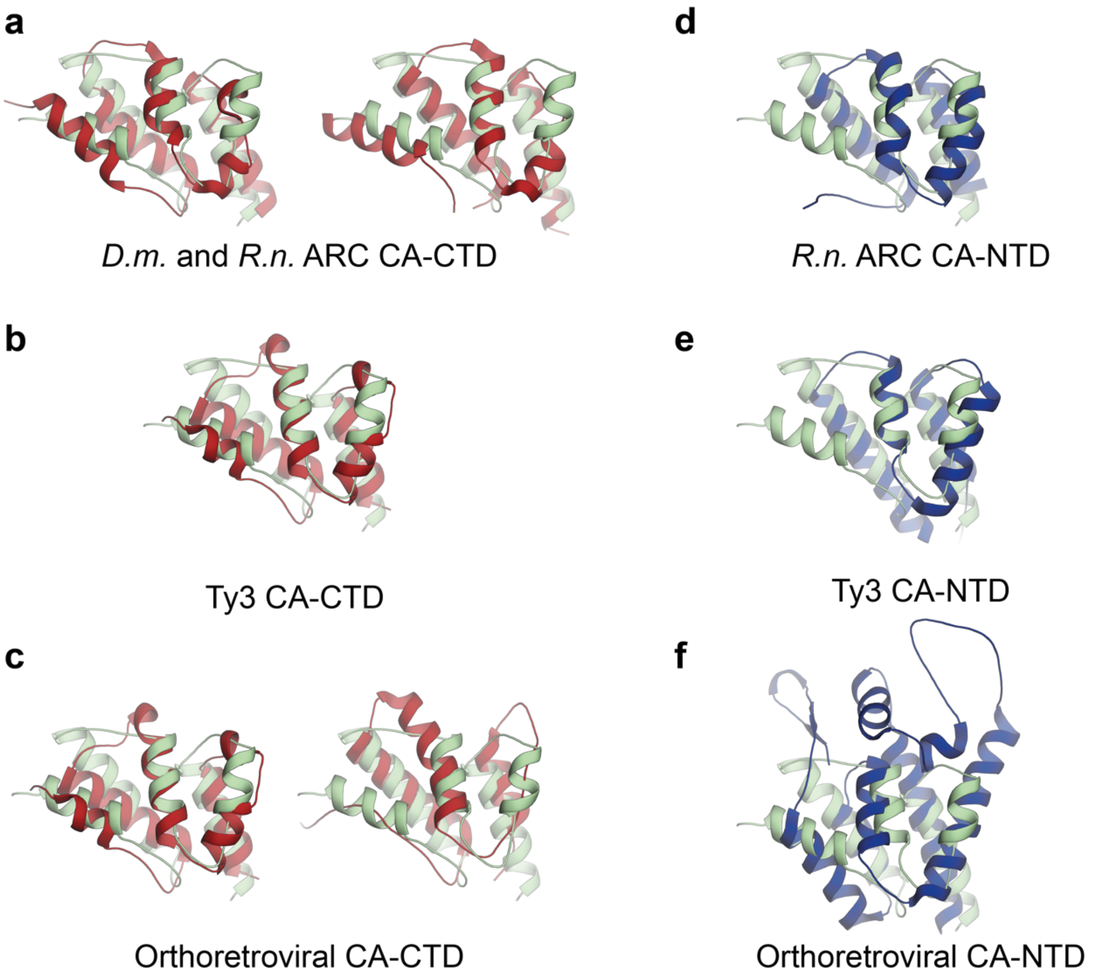

g

Panel	CA domain	PDB code	Z score	rmsd (Å)	#Cα aligned	seqID (%)
**a**	CTD	6S7X	8.6	2.4	82	15
	CTD	4X3X	8.2	2.6	78	17
**b**	CTD	6R23	7.7	3.2	81	14
**c**	CTD	6GZA	7.8	2.7	81	9
	CTD	6SSK	5.3	3.2	78	9
**d**	NTD	4X3I	5.8	2.2	61	10
**e**	NTD	6R22	6.1	2.2	66	11
**f**	NTD	5UP4	4.9	3.3	73	5

Supplementary Fig. 1. Structural similarity of p18m with Gag CA-CTD and CA-NTD domains. (**a-f**) Pairwise DALI 3D Cα structural alignment of p18m with (**a**) ARC proteins; *D. melanogaster* ARC1 CA-CTD & *R. norvegicus* ARC1 CA-CTD, (**b**) Transposon Gag; Ty3 CA-CTD, (**c**) orthoretroviral Gag; MLV CA-CTD & HERV-K CA-

CTD (**d**) *R. norvegicus* ARC1 CA-NTD, (**e**) Ty3 CA-NTD and (**f**) HIV-1 CA-NTD. In each panel, the cartoon of the p18m backbone is shown in pale green and the backbone of the aligned structures ate shown in red (CA-CTDs) and blue (CA-NTDs). (**g**) PDB IDs, DALI Z scores, RMSD, number of aligned residues, and sequence identities for 3D Cα alignments.

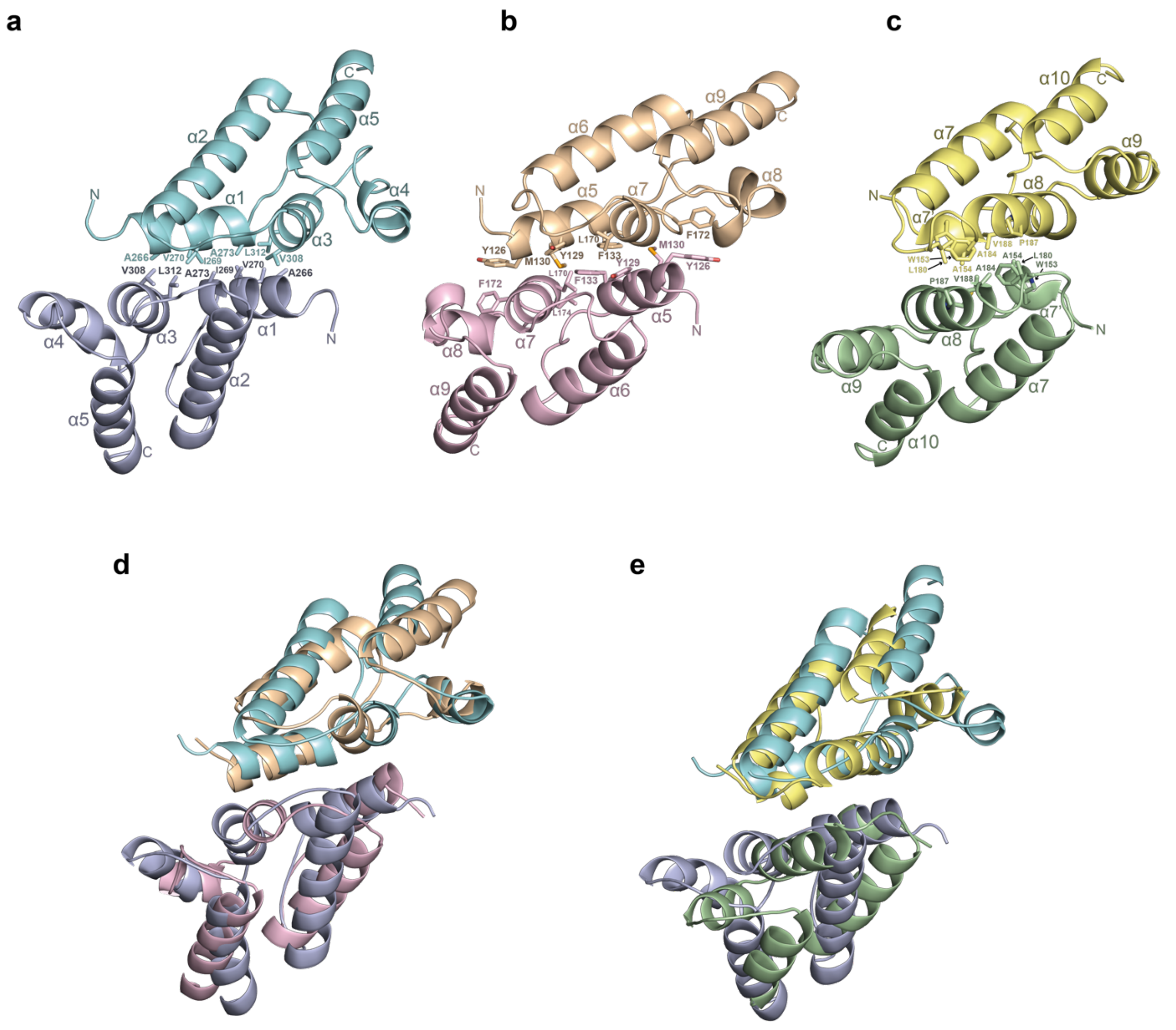

Supplementary Fig. 2. Comparison of CTD-CTD dimer interfaces. Dimer structures of (**a**) p18m, (**b**), dARC1 CA-CTD (PDB ID: 6S7X) and (**c**) RSV CA-CTD (PDB ID: 3G21) are shown in cartoon representation. Helices are numbered sequentially from the N- to C-terminus, α1 - α5 of p18m equate to α5 - α9 of DARC1 and α7' - α10 of RSV CA to account for the N-terminal domains of dARC1 and RSV-CA. The structures are aligned relative to p18m and viewed down the 2-fold symmetry axis of the dimer interface. In each dimer, residues that contribute to the continuous apolar networks at the interface are labelled and shown in stick representation. (**d** & **e**) Structural alignments of the p18m dimer with the dARC1 CTD dimer (**d**) and RSV CA-CTD dimer (**e**). Views and protein backbone in cartoon representation, colours are as in **a**-**c**.

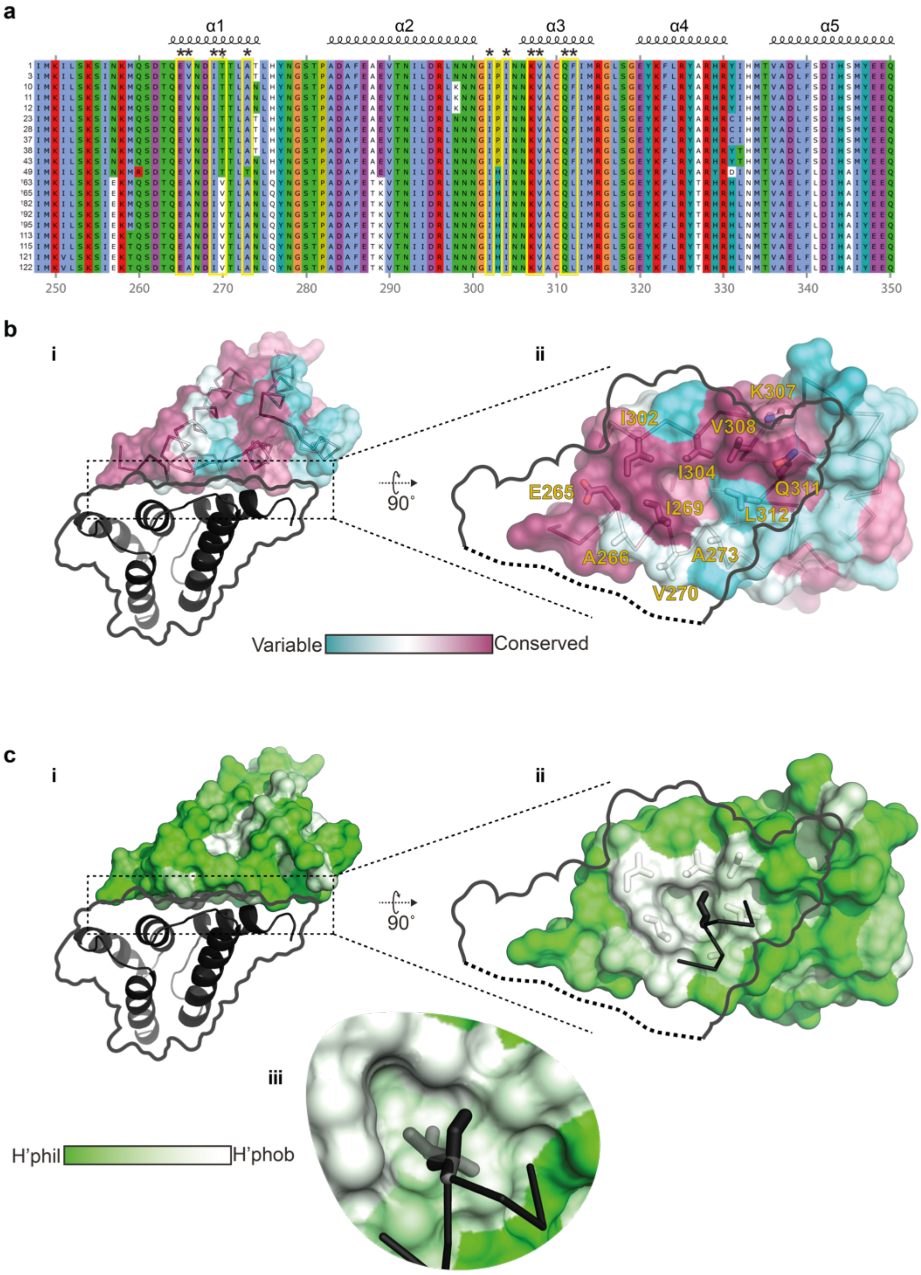

Supplementary Fig. 3. Sequence conservation at the p18m Dimer-1 interface. (a) Sequence alignment of the p18m region of Gag from the Ty1 elements of *Saccharomyces* spp, coloured according to the Clustal scheme[1]. Shown are 20 sequences from a larger alignment of 125 Ty1 elements mined from a BLAST search of the SGD (https://www.yeastgenome.org/). The 20 shown sequences were selected to proportionally represent the diversity found at the Dimer-1 interface. The numbering corresponds to the position in the larger 125 sequence alignment (https://crick.figshare.com/articles/dataset/Cottee_Supplementary_datafile1_Ty1p18_

alignment_txt/15060366). Sequences annotated with (**†)** are identical to the p18m used in this study. The secondary structure of p18m is indicated above, and residues at the dimer-1 interface are indicated by asterisks and yellow boxes. (**b**) Sequence conservation at the p18m Dimer-1 interface. (i) Chain A is outlined and shown as a backbone cartoon. Chain B is shown as a ribbon with a molecular surface, coloured as cyan through white to purple according to increased conservation among Ty1 sequences. (ii), view of the dimer interface, looking into chain B. Interfacing residues are shown as sticks and are largely conserved. The central residue I269 is universally conserved as are the most hydrophobic parts of the interface. (**c**) Hydrophobicity at the p18m dimer interface. Views (i) and (ii) are the same as in B with chain B shown as a surface, coloured according to increased residue hydrophobicity, green to white. At the centre of the interface a hydrophobic patch is formed by the residues shown as sticks, most of which are highly conserved. At its centre, a cavity surrounds I269 from chain A (shown as black sticks) (iii) Closeup view of the hydrophobic cavity. Several rotamers of I269 (shades of grey) are likely acceptable in the cavity. Additionally, as suggested by the I269F substitution (**Fig. 4 & 5**), the cavity could accommodate larger residues.

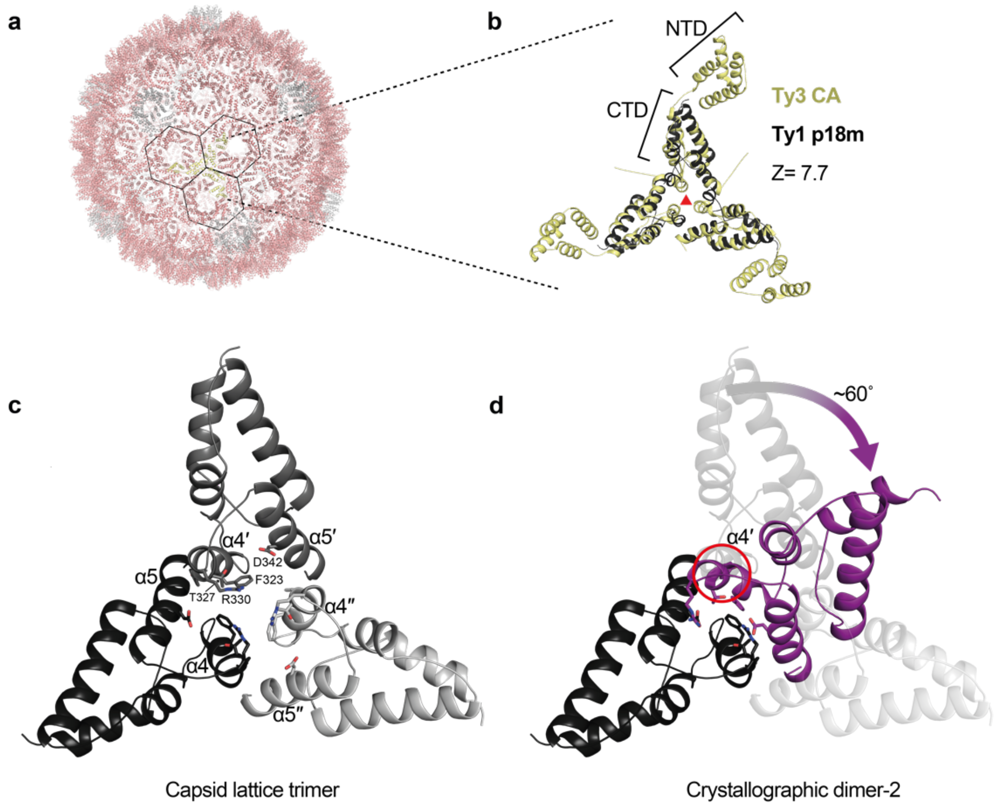

Supplementary Fig. 4. Dimer-2 interface and the Ty1 Gag particle 3-fold axis. (**a**) The Ty3 CA T=9 icosahedral particle (PDB ID: 6R24). The backbone of each Ty3 CA molecule is shown in cartoon representation. Chains in hexameric capsomeres are coloured red, chains forming pentamer capsomeres are grey. Three adjacent hexamers surrounding a 3-fold axis are highlighted by the hexagons with the chains at the intercapsomere 3-fold coloured yellow. (**b**) Superposition of p18m and Ty3-CA. The three Ty3 CA monomers at the trimer interface are shown in yellow cartoon. p18m (black cartoon) superposes onto the CTD of each copy of Ty3 CA (DALI Z score; 7.7) approximating the trimeric arrangement of Ty1 CA-CTD in an assembled VLP. (**c**) Close up of the modelled Ty1 CA-CTD trimer. The backbone of the three p18m monomers are shown in cartoon, light to dark grey. Residues that make contacts across the crystallographic Dimer-2 interface (**Fig. 2**) are shown as sticks. They present towards the trimer interface of the modelled structure and labelled for one

p18m copy. (**d**) The p18m crystallographic Dimer-2 interface. The two copies of p18m at the Dimer-2 interface are shown in black and purple. Positions of chains in the trimeric arrangement are shown translucent. Transition from the trimer to dimer arrangement requires the rotation of one chain around α4 (red circled), by approximately 60˚.

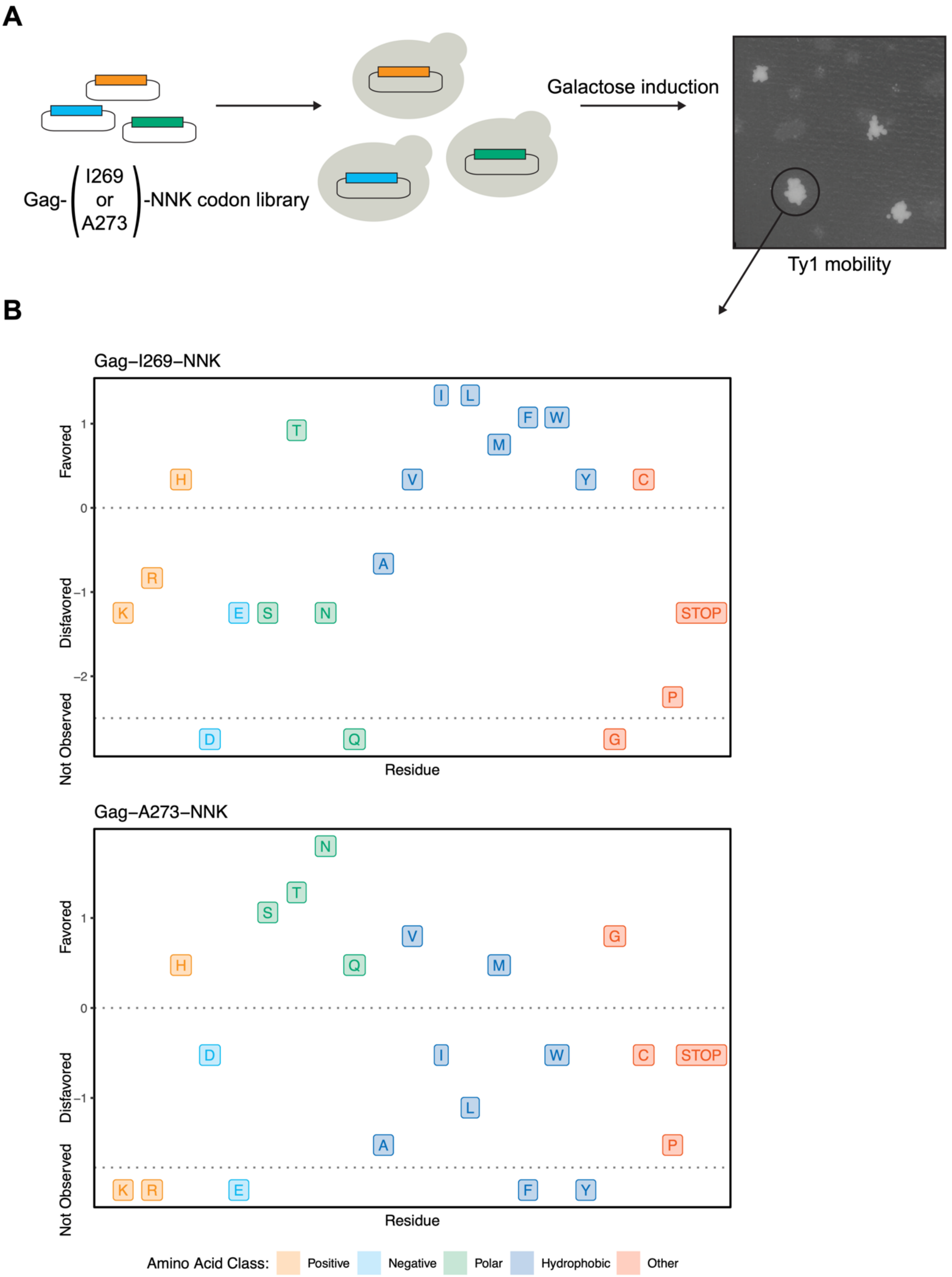

Supplementary Fig. 5. Dimer-1 interface mutagenesis screen. (**a**) Schematic illustrating two NNK codon screens (N = A/C/T/G, K = G/T) carried out at Gag-269 and Gag-273. This strategy randomizes the amino acids at either position, encoding all 20

amino acids while reducing premature stop codons. Codon libraries were generated in a pGTy1*his3-AI* plasmid and mutants with detectable papillation were sequenced. (**b**) Log_2-transformed observed/expected ratios for each amino acid encoded in the NNK library. Positive values are overrepresented compared to the codon make-up of the NNK library, negative values are observed less frequently than expected. Residues are coloured by biochemical property. The NNK library encodes for 1 stop codon. Several residues were never observed at a given position. 76 papillation-competent clones were sequenced from the Gag-269-NNK library and 46 from the Gag-273-NNK library. Follow-up validation of several mutants (**Supplementary Table 4**) confirmed that overrepresented mutants usually had high retromobility whereas underrepresented mutants were more likely to have low retromobility.

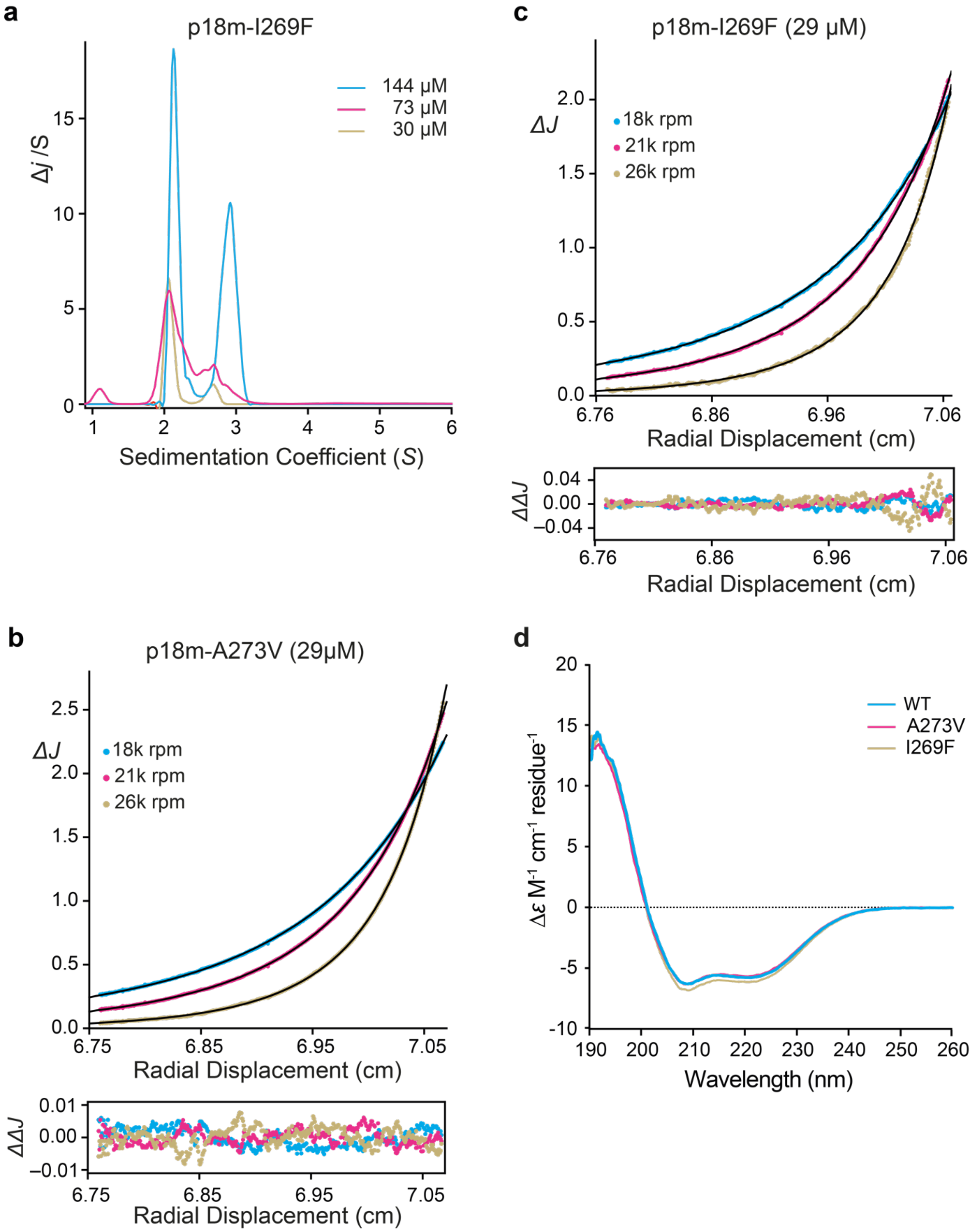

Supplementary Fig. 6. p18m interface mutants A273V and I269F do not disrupt Dimer-1 or protein folding. (**a**) C(S) distributions derived from sedimentation velocity data recorded from p18m-I269F at 30 μM (wheat), 73 μM (magenta) and 144 μM (cyan). The curves are the distribution of the sedimentation coefficients that best fit the

sedimentation data (RMSD 0.006 - 0.013). (**b-c**) Multi-speed sedimentation equilibrium profiles determined from interference data collected on p18m-A273V and p18m-I269F at 29 µM. Data was recorded at the speeds indicated. The recorded data is shown as points and the solid lines represent the global best fit using a monomer-dimer-tetramer model. For p18m-A273V, $K_D^{(1\text{-}2)}$ = 0.34 µM, $K_D^{(2\text{-}4)}$ = 51.8 µM; reduced χ^2 = 0.53. For p18m-I269F, $K_D^{(1\text{-}2)}$ = 0.74 µM, $K_D^{(2\text{-}4)}$ = 45.3 µM; reduced χ^2 = 2.58. The lower panels show the residuals to the fits. Source data for **a** - **c** are provided in the Source Data file. **(d**) Far UV CD spectra (260 to 190 nm) of p18m, p18m-A273V and p18m-I269F recorded at 10 °C.

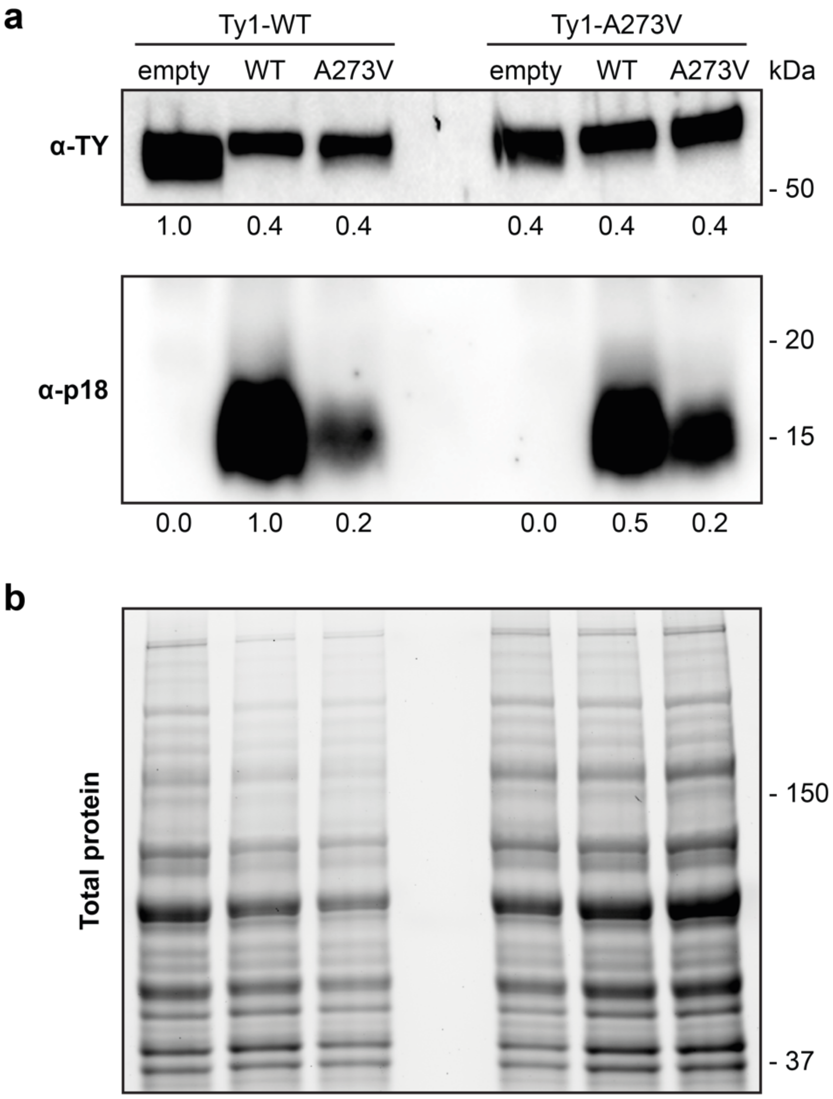

Supplementary Fig. 7. Protein expression in strains used for heterotypic interactions (a) Immunoblots of protein extracts prepared from galactose-induced yeast cells expressing the indicated single-copy chromosomal Ty1 and plasmid-borne p18m$_{AUG1}$ mutants. Extracts were immunoblotted with TY-tag antibody to detect Gag or p18 antibody to detect chromosomal p22/p18 and ectopic p18m$_{AUG1}$. Numbers below immunoblots indicate relative levels of immunoreactive species normalized to total protein load. Migration of molecular weight standards are shown alongside the immunoblots, a representative image of at least 3 replicates is shown. Images of the whole gel immunoblots are provided in the Source Data file. The chromosomal Ty1-A273V is not more highly expressed and does not produce detectable p22/p18, see

also **(Fig. 6g & Supplementary Table 1).** Ty1-WT strains used: empty (DG4296), WT (DG4297), A273V (DG4298). Ty1-A273V strains used: empty (DG4279), WT (DG4280), A273V (DG4281). (**b**) Total protein loading control. Representative image of at least 3 replicates is shown.

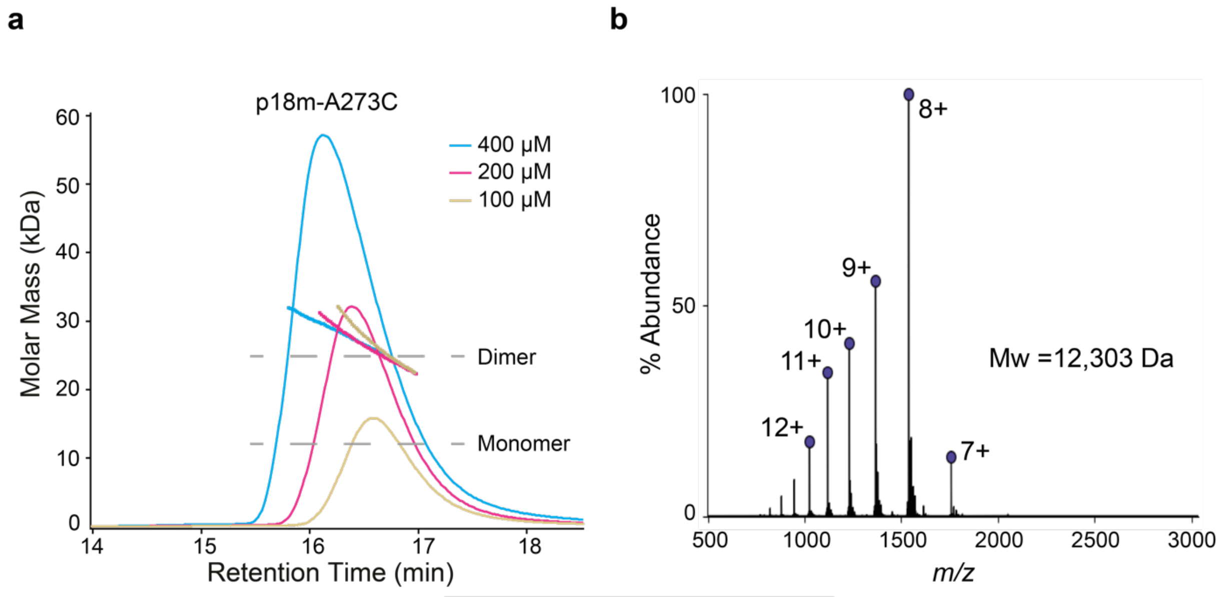

Supplementary Fig. 8. p18m-A273C mutation is tolerated at the Dimer-1 interface. (**a**) SEC-MALLS analysis of p18m-A273C interface mutant. Sample loading concentrations were 400 μM (cyan), 200 μM (magenta) and 100 μM (wheat). The differential refractive index (dRI) is plotted against column retention time and the molar mass, determined at 1-second intervals throughout the elution of each peak, is plotted as points. The monomer and dimer molecular mass is indicated with the grey dashed lines. (**b**) ESI-MS analysis of p18m-A273C. Only ions corresponding to the monomer molecular mass (12,303 Da) are found in the spectrum.

Supplementary Table 1. Retromobility frequencies.

Data appears in figure:	Strain	Label	Retromobility Frequency (x10[7])	Std Dev (x10[7])	Fold decrease	*p*-value[a]
Fig. 1	DG3739	Empty	400,221.73	32,106.14	1	**2.74E-07**
	DG4162	p18	3.93	0.93	101,905	Reference
	DG4147	p18m$_{AUG1}$	4.26	1.76	94,025	0.75
	DG4146	p18m$_{AUG2}$	17,808.22	2,770.62	22	**1.36E-05**
Fig. 4	DG3735	Gag-WT	109,393.06	15,839.80	1	Reference
	DG4342	Gag-A273V	198,115.43	34,470.77	(↑) 1.8[b]	**0.0034**
	DG4341	Gag-I269F	42,443.73	6,485.42	2.6	**0.0002**
Fig. 4	DG3739	Empty	357,408.24	52,457.09	1	**4.08E-17**
	DG4147	p18m$_{AUG1}$-WT	4.42	2.17	80,875	Reference
	DG4165	p18m$_{AUG1}$-A273V	5.03	0.68	71,030	0.59
	DG4340	p18m$_{AUG1}$-I269F	2.49	1.26	143,345	0.12
Fig. 6[c]	DG4296	Ty1/empty	911.85	115.44	1.0	5.49E-06
	DG4297	Ty1/p18m$_{AUG1}$	34.35	18.04	26.5	Reference
	DG4298	Ty1/p18m$_{AUG1}$-A273V	405.15	41.75	2.3	3.39E-06
	DG4279	Ty1-A273V/empty	19,450.55	2,847.21	1.0	1.64E-05
	DG4280	Ty1-A273V/p18m$_{AUG1}$	1,594.16	354.64	12.2	Reference
	DG4281	Ty1-A273V/p18m$_{AUG1}$-A273V	5,586.10	952.50	3.5	2.25E-04
			**Relative restriction[d]**	**Std Dev**		
		Ty1/p18m$_{AUG1}$	100%			Reference
		Ty1-A273V/p18m$_{AUG1}$	46%	9%		**0.039**
		Ty1/p18m$_{AUG1}$-A273V	65%	6%		**0.013**
		Ty1-A273V/p18m$_{AUG1}$-A273V	100%			Reference

[a] Calculated by two-sided Student's *t*-test; $p < 0.05$ are bolded.
[b] Arrow indicates fold increase, rather than fold decrease.
[c] The retromobility frequencies underlying the relative restriction numbers presented in Fig. 6 are included here. Although Ty1 and Ty1-A273V chromosomal integrations are independently derived, Ty1-A273V mobility appears to be hypermorphic based on immunoblot analysis (Supplementary Fig. 7), as observed previously[2].
[d] Relative restriction is calculated as the percentage of homotypic fold restriction.

Supplementary Table 2. p18m Data collection, phasing and refinement statistics.

	p18m$_{AUG2}$ (Se, peak)	p18m$_{AUG2}$ (Se, hrem)	p18m$_{AUG2}$ (Se, infl)	p18m$_{AUG1}$	p18m$_{AUG2}$-A273V
**Data collection**					
Space group	P6$_5$22	P6$_5$22	P6$_5$22	P6$_5$22	P6$_5$22
Cell dimensions					
a, b, c (Å)	280.89, 280.89,40.41	281.17, 281.17,40.50	281.58, 281.58, 40.61	282.12, 282.12, 39.97	279.87, 279.87, 39.92
α, β, γ (°)	90, 90, 120	90, 90, 120	90, 90, 120	90, 90, 120	90, 90, 120
Wavelength (Å)	0.97928	0.97281	0.97943	0.97954	0.97951
Resolution (Å)	140.45-3.12 (3.33-3.12)[†]	81.17-3.41 (3.68-3.41)	92.17-3.50 (3.83-3.50)	244.4-2.80 (2.95 2.80)	91.61-3.53 (3.59-3.53)
Unique reflections	17,434 (5059)	13,524 (2736)	12,627 (2927)	23,876 (3390)	11,994 (596)
R_{meas} (%)	[‡]24.6 (131.3)	[‡]23.0 (100.4)	[‡]23.1 (97.0)	10.1 (391.2)	14.6 (237.9)
R_{pim} (%)	[‡]3.1 (16.7)	[‡]4.2 (19.2)	[‡]4.2 (17.7)	1.6 (62.7)	3.0 (43.0)
$CC_{1/2}$	0.983 (0.760)	0.999 (1.000)	0.999 (1.000)	1.000 (0.491)	1.000 (0.626)
$I/σ(I)$	24.5 (6.8)	16.5 (5.0)	16.0 (5.2)	21.6 (1.2)	17.7 (1.7)
Completeness (%)	100.0 (100.0)	100.0 (100.0)	100.0 (100.0)	100.0 (100.0)	100.0 (98.5)
Multiplicity	112.8 (115.8)	55.5 (56.5)	55.4 (56.7)	38.7 (38.6)	54.0 (30.3)
Anom Multiplicity	61.0 (60.7)	30.1 (29.8)	30.1 (29.8)	-	-
Overall CC_{anom}	0.922	0.714	0.515	-	-
Significant anomalous signal to: (AIMLESS) (Å)	3.76	5.03	5.79	-	-
**Phasing**					
No. sites (found/expected)	8/6				
CC_{all}/CC_{weak} (ShelxD)	47.48/32.76				
Estim. mean FOM (ShelxE)	0.614 (140.45-3.12 Å)				
Pseudo-free CC (ShelxE) (%)	65.37				

[†]Values in parenthesis refer to the highest resolution shell, [‡]Values quoted for within I^+/I^-, [§]Friedel pairs separated.

Supplementary Table 2. p18m Data collection, phasing and refinement statistics (continued).

	p18m$_{AUG2}$ (Se, peak)	p18m$_{AUG2}$ (Se, h-rem)	p18m$_{AUG2}$ (Se, infl)	p18m$_{AUG1}$	p18m$_{AUG2}$-A273V
**Refinement**	Phenix-anom			Refmac	Phenix
Resolution (Å)	91.98-3.12 (3.21-3.12)†			141.46-2.80 (2.87-2.80)	91.61-3.53 (3.80-3.53)
Refl working/free	§31754/1663 (2430/152)			22603/1259 (1651/98)	11953/630 (2209/104)
R_{work}/R_{free}/Test set size (%)	23.3/25.8/5.2 (34.0/38.8/5.9)			25.6/26.4/5.3 (39.7/37.6/5.6)	22.5/27.2/5.3 (29.0/36.3/4.5)
No residues/atoms					
Protein	274/2211			271/2189	270/2186
Ligands	0			0	0
Water	0			0	0
B-factors ($Å^2$)					
Wilson	58.87			95.49	171.93
Protein	94.90			145.56	187.56
Ligands	-			-	-
Water	-			-	-
Overall	94.90			145.46	187.56
Geometry					
RMSD Bond lengths (Å)	0.001			0.004	0.002
RMSD Bond angles (°)	0.373			1.29	0.484
Ramachandran Outliers (%)	0.00			1.13	0.00
Ramachandran Favoured (%)	99.25			95.09	93.18
Molprobity score (N number/percentile)	1.09 (2019,100th)			1.91 (4482, 99th)	1.69 (342, 100th)

†Values in parenthesis refer to the highest resolution shell, ‡Values quoted for within I^+/I^-, §Friedel pairs separated.

Supplementary Table 3. p18m Sedimentation data.

Hydrodynamic parameters			
**Protein**	p18m	p18m-A273V	p18m-I269F
^{a}v (mL.g^{-1})	0.722	0.723	0.721
$^{b}\rho$ (g.mL^{-1})	1.013	1.013	1.013
$^{c}\eta$ (x10^{2}) (g^{-1} cm^{-1} s^{-1})	1.043	1.043	1.043
$^{d}M_r$ (g mole^{-1})	12,272	12,300	12,306
$^{e}\varepsilon_{280}$ (M^{-1} cm^{-1})	5,200	5,200	5,200
$^{f}J_{inc}$ (M^{-1}.cm^{-1})	33,748	33,825	33,842

[a]Protein partial specific volume; [b]Buffer density; [c]Buffer viscosity [d]Molar mass calculated from the protein sequence; [e]Molar absorbance extinction coefficient; [f]Molar fringe increment.

Supplementary Table 3. p18m Sedimentation data (continued).

Sedimentation velocity			
**p18m**			
C (μM)	42	82	165
[a]$S_{20,w}$ (x10^{13}) sec (S_{slow})	2.35	2.31	2.30
[b]θ (S_{slow})	1.0	0.68	0.49
$S_{20,w}$ (x10^{13}) sec (S_{fast})	/	2.90	3.09
θ (S_{fast})	0	0.32	0.51
[c]$S_{20,w}$ (x10^{13}) sec ($w_{averaged}$)	2.35	2.50	2.70
[d]f/f_0 C(S)	1.27	1.24	1.26
[e]M_w C(S) kDa (S_{slow})	22.6	21.4	21.7
M_w C(S) kDa (S_{fast})	/	30.0	33.7
[f]rmsd C(S)	0.004	0.010	0.016
**p18m-A273V**			
C (μM)	40	86	181
$S_{20,w}$ (x10^{13}) sec (S_{slow})	2.35	2.27	2.31
θ (S_{slow})	1.0	0.56	0.43
$S_{20,w}$ (x10^{13}) sec (S_{fast})	/	2.89	3.18
θ (S_{fast})	0	0.44	0.57
$S_{20,w}$ (x10^{13}) sec ($w_{averaged}$)	2.42	2.54	2.80
f/f_0 C(S)	1.27	1.26	1.19
M_w C(S) kDa S_{slow})	22.9	21.2	20.1
M_w C(S) kDa S_{fast})	/	30.5	32.4
rmsd C(S)	0.009	0.010	0.025
**p18m-I269F**			
C (μM)	30	73	144
$S_{20,w}$ (x10^{13}) sec (S_{slow})	2.25	2.33	2.33
θ (S_{slow})	0.83	0.74	0.52
$S_{20,w}$ (x10^{13}) sec (S_{fast})	2.87	2.96	3.13
θ (S_{fast})	0.17	0.26	0.48
$S_{20,w}$ (x10^{13}) sec ($w_{averaged}$)	2.35	2.49	2.71
f/f_0 C(S)	1.48	1.25	1.25
M_w C(S) kDa (S_{slow})	26.4	21.7	21.8
M_w C(S) kDa (S_{fast})	38.2	31.1	33.8
rmsd C(S)	0.006	0.006	0.013

[a]$S_{20,w}$ value (x10^{13}) sec for each resolved species in the C(S) function; [b]Fraction of each resolved species derived from integration of the best fit C(S) function; [c]Weight averaged sedimentation coefficient determined from integration of the peak envelope in the best fit C(S) function; [d]The weight-averaged frictional ratio from the best fit C(S) distribution function; [e]Weight averaged molecular weight from C(S) distribution function; [f]root mean square deviation of the best fit C(S) model.

Supplementary Table 3. p18m Sedimentation data (continued).

Sedimentation equilibrium				
**p18m**				
C (μM)	30	64	122	30-122
[a]M_w kDa	31.6	38.5	39.6	31.6 - 39.6
[b]$K_D^{(1\text{-}2)}$ (μM)	0.82	0.67	0.57	0.73
[c]$K_D^{(2\text{-}4)}$ (μM)	97.5	42.4	41.5	43.2
[d]rmsd	0.002	0.004	0.011	0.004 - 0.011
[e]χ^2				2.22
**p18m-A273V**				
C (μM)	29	/	122	29-122
M_w kDa	35.8		41.9	35.8 - 41.9
$K_D^{(1\text{-}2)}$ (μM)	0.46		0.25	0.34
$K_D^{(2\text{-}4)}$ (μM)	48.6		40.6	51.8
rmsd	0.002		0.004	0.003 - 0.005
χ^2				0.53
**p18m-I269F**				
C (μM)	29	61	/	29-61
M_w kDa	29.9	36.1		29.9 – 36.1
$K_D^{(1\text{-}2)}$ (μM)	0.89	0.74		0.74
$K_D^{(2\text{-}4)}$ (μM)	38.8	31.7		45.3
rmsd	0.01	0.004		0.004 - 0.01
χ^2				2.58

[a]weight averaged molecular weight derived from Global analysis of individual samples using single species model; [b]monomer-dimer equilibrium dissociation constant determined from a global fit using three concentrations and three speeds to a monomer-dimer-tetramer self-association model; [c]Dimer-tetramer equilibrium dissociation constant derived from a monomer-dimer-tetramer self-association model; [d]root mean square deviation observed for each multi-speed sample when fitted individually and globally; [e]global reduced chi-squared from combined fitting of all multispeed data.

Supplementary Table 4. Summary of mutations analysed.

Organism	Construct	Mutation	Solubility	SEC-MALLS	AUC
E. coli	p18m$_{AUG2}$	WT	High	Yes	Yes
	p18m$_{AUG2}$	A273V	High	Yes	Yes
	p18m$_{AUG2}$	A273D	Insoluble		
	p18m$_{AUG2}$	A273Q	Insoluble		
	p18m$_{AUG2}$	A273M	Insoluble		
	p18m$_{AUG2}$	A273C*	Medium	Yes	
	p18m$_{AUG2}$	I269S	Insoluble		
	p18m$_{AUG2}$	I269K	Insoluble		
	p18m$_{AUG2}$	I269P	Insoluble		
	p18m$_{AUG2}$	I269F	Medium	Yes	Yes
	p18m$_{AUG2}$	F323S	High	Yes	
			**Expression**	**Retromobility**	**Restriction**
S. cer	p22 FL	WT	High		Strong
	p22-6His FL	WT	High		Strong
	p18-6His FL	WT	High		Strong
	p22 FL	A285D-T288D	High		Strong
	p22 FL	V308E-L312E	Low		None
	p22 FL	I269S-V270S	Low		None
	p22 FL	I269E-V270E	Low		None
	p22 FL	A273V	Medium		Strong
	p18m$_{AUG2}$	WT	Low		Medium
	p18m$_{AUG1}$	WT	High		Strong
	p18m$_{AUG1}$	L312S	Low		None
	p18m$_{AUG1}$	I269S	Low		None
	p18m$_{AUG1}$	I269P	Low		None
	p18m$_{AUG1}$	I269F	High		Strong
	p18m$_{AUG1}$	A273M	Low		None
	p18m$_{AUG1}$	A273V	High		Strong
	p18m$_{AUG1}$	A273S	High		Strong
	p18m$_{AUG1}$	A273C	High		Strong
	p18m$_{AUG1}$	F323S	High		Strong
	p18m$_{AUG1}$	F323D	High		Strong
	Gag	L312S	High	High	
	Gag	L312F	High	High	
	Gag	V270T	High	High	
	Gag	V270S	High	High	
	Gag	V270T-L312F	High	High	
	Gag	I269S-V270S-L312S	Low	Low	
	Gag	I269S	Low	Low	
	Gag	I269T	Low	High	
	Gag	I269P	Low	Low	
	Gag	I269F	High	High	
	Gag	I269L	High	High	
	Gag	A273H	Medium	Low	
	Gag	A273M	Medium	Low	
	Gag	A273C	High	High	
	Gag	A273V	High	High	
	Gag	F323S	High	Low	
	Gag	F323D	High	Low	

*Mass spectrometric analysis indicates no disulphide linked chains

Supplementary Table 5. Yeast strains.

Strain	Genotype	Plasmids	Source
DG3582	*MATα gal3 his3-Δ200hisG trp1-1* ura3* Ty1-less		[3]
DG3713	DG3582 +1 Ty1*his3-AI*		[2]
DG3719	DG3582 +1 Ty1*his3-AI-A273V*		[2]
DG3735	DG3582	pBDG1534	This study
DG3739	DG3582	pBDG1534, pBDG1293	[3]
DG3774	DG3582	pBDG1534, pBDG1565	[3]
DG4146	DG3582	pBDG1534, pBDG1645	This study
DG4147	DG3582	pBDG1534, pBDG1646	This study
DG4162	DG3582	pBDG1534, pBDG1656	This study
DG4165	DG3582	pBDG1534, pBDG1657	This study
DG4279	DG3719	pBDG1293	This study
DG4280	DG3719	pBDG1646	This study
DG4281	DG3719	pBDG1657	This study
DG4292	DG3582	pBDG1646	This study
DG4296	DG3713	pBDG1293	This study
DG4297	DG3713	pBDG1646	This study
DG4298	DG3713	pBDG1657	This study
DG4340	DG3582	pBDG1534, pBDG1736	This study
DG4341	DG3582	pBDG1745	This study
DG4342	DG3582	pBDG1746	This study
DG4348	DG3582	pBDG1749	This study
DG4349	DG3582	pBDG1750	This study
DG4350	DG3582	pBDG1534, pBDG1747	This study
DG4351	DG3582	pBDG1534, pBDG1748	This study

Supplementary Table 6. Yeast plasmids.

Plasmid	Description	Markers	Source
pBDG1293	p*GAL*-Yes2	*URA3/2μ*	Invitrogen
pBDG1534	pGTy1*his3-AI*	*TRP1/CEN*	[3]
pBDG1565	pBDG1293-p22	*URA3/2μ*	[3]
pBDG1645	pBDG1293-p18$_{AUG2\text{-}355}$-6xHis	*URA3/2μ*	This study
pBDG1646	pBDG1293-p18$_{AUG1\text{-}355}$-6xHis	*URA3/2μ*	This study
pBDG1656	pBDG1293-p18-6xHis	*URA3/2μ*	This study
pBDG1657	pBDG1646-*A273V*	*URA3/2μ*	This study
pBDG1736	pBDG1646-*I269F*	*URA3/2μ*	This study
pBDG1745	pBDG1534-*GAG-I269F*	*TRP1/CEN*	This study
pBDG1746	pBDG1534-*GAG-A273V*	*TRP1/CEN*	This study
pBDG1747	pBDG1646-*F323S*	*URA3/2μ*	This study
pBDG1748	pBDG1646-*F323D*	*URA3/2μ*	This study
pBDG1749	pBDG1534-*GAG-F323S*	*TRP1/CEN*	This study
pBDG1750	pBDG1534-*GAG-F323D*	*TRP1/CEN*	This study

Supplementary Table 7. p18m cloning and mutagenic primers

Construct		Oligos (5′-3′)*#
Synthetic gene sequence for Ty1 (P08405)	-	ATGAAAATCCTGAGCAAAAGCATCGAGAAAATGCAGAGCGATACCCAAG AAGCAAACGATATTGTTACCCTGGCAAATCTGCAGTATAACGGTAGTAC ACCGGCAGATGCATTTGAAACCAAAGTGACCAACATTATTGATCGCCTG AATAACAACGGCATCCACATTAATAACAAAGTTGCCTGTCAGCTGATTA TGCGTGGTCTGAGCGGTGAATACAAATTTCTGCGTTATACCCGTCATCG TCATCTGAATATGACCGTTGCAGAACTGTTTCTGGATATTCATGCCATC TATGAAGAACAGCAGGGTAGCCGTAAT
pET22b_M249	FWD	GAAGGAGATATACATATGAAAATCCTGAGCAAAAG
pET22b_M259	FWD	GAAGGAGATATACATATGCAGAGCGATACCCAAG
pET22b_N355	REV	GTGGTGGTGGTGGTGCTCGAGCGGATTACGGCTACCCTG
Mutagenic Primer Pairs		
I269F	FWD	CGATACCCAAGAAGCAAACGATTTTGTTACCCTGGCAAATCTGCAGT
	REV	ACTGCAGATTTGCCAGGGTAACAAAATCGTTTGCTTCTTGGGTATCG
I269S	FWD	CGATACCCAAGAAGCAAACGATAGCGTTACCCTGGCAAATCTGCAGT
	REV	ACTGCAGATTTGCCAGGGTAACGCTATCGTTTGCTTCTTGGGTATCG
I269K	FWD	CGATACCCAAGAAGCAAACGATAAAGTTACCCTGGCAAATCTGCAGT
	REV	ACTGCAGATTTGCCAGGGTAACTTTATCGTTTGCTTCTTGGGTATCG
A273V	FWD	GCAAACGATATTGTTACCCTGGTGAATCTGCAGTATAACGGTAGTAC
	REV	GTACTACCGTTATACTGCAGATTCACCAGGGTAACAATATCGTTTGC
A273C	FWD	GCAAACGATATTGTTACCCTGTGCAATCTGCAGTATAACGGTAGTAC
	REV	GTACTACCGTTATACTGCAGATTGCACAGGGTAACAATATCGTTTGC
A273Q	FWD	GCAAACGATATTGTTACCCTGCAGAATCTGCAGTATAACGGTAGTAC
	REV	GTACTACCGTTATACTGCAGATTCTGCAGGGTAACAATATCGTTTGC
A273D	FWD	GCAAACGATATTGTTACCCTGGATAATCTGCAGTATAACGGTAGTAC
	REV	GTACTACCGTTATACTGCAGATTATCCAGGGTAACAATATCGTTTGC
A273M	FWD	GCAAACGATATTGTTACCCTGATGAATCTGCAGTATAACGGTAGTAC
	REV	GTACTACCGTTATACTGCAGATTCATCAGGGTAACAATATCGTTTGC
F323S	FWD	GTGGTCTGAGCGGTGAATACAAAAGCCTGCGTTATACCCGTCATCGTCA
	REV	TGACGATGACGGGTATAACGCAGGCTTTTGTATTCACCGCTCAGACCAC

*Restriction sites used for cloning are underlined; #Mutagenized codons are highlighted and underlined.

Supplementary References

1. Larkin MA*, et al.* Clustal W and Clustal X version 2.0. *Bioinformatics* **23**, 2947-2948 (2007).

2. Tucker JM, Larango ME, Wachsmuth LP, Kannan N, Garfinkel DJ. The Ty1 Retrotransposon Restriction Factor p22 Targets Gag. *PLoS genetics* **11**, e1005571 (2015).

3. Saha A*, et al.* A trans-dominant form of Gag restricts Ty1 retrotransposition and mediates copy number control. *Journal of virology* **89**, 3922-3938 (2015).
