# Supplementary material for: Structure of a Ty1 restriction factor reveals the molecular basis of transposition copy number control
Source: Nat Commun. 2021 Sep 22;12:5590. doi: 10.1038/s41467-021-25849-0 (PMC8458377; doi:10.1038/s41467-021-25849-0)
Supplement: Supplementary file 2 — Reporting summary [file 41467_2021_25849_MOESM2_ESM.pdf]

nature research

Corresponding author(s): Ian A Taylor & David J Garfinkel

Last updated by author(s): Aug 24, 2021

Reporting Summary

Nature Research wishes to improve the reproducibility of the work that we publish. This form provides structure for consistency and transparency in reporting. For further information on Nature Research policies, see our Editorial Policies and the Editorial Policy Checklist.

Statistics

For all statistical analyses, confirm that the following items are present in the figure legend, table legend, main text, or Methods section.

n/a	Confirmed	
☐	☒	The exact sample size (n) for each experimental group/condition, given as a discrete number and unit of measurement
☐	☒	A statement on whether measurements were taken from distinct samples or whether the same sample was measured repeatedly
☐	☒	The statistical test(s) used AND whether they are one- or two-sided<br>*Only common tests should be described solely by name; describe more complex techniques in the Methods section.*
☒	☐	A description of all covariates tested
☒	☐	A description of any assumptions or corrections, such as tests of normality and adjustment for multiple comparisons
☐	☒	A full description of the statistical parameters including central tendency (e.g. means) or other basic estimates (e.g. regression coefficient) AND variation (e.g. standard deviation) or associated estimates of uncertainty (e.g. confidence intervals)
☐	☒	For null hypothesis testing, the test statistic (e.g. F, t, r) with confidence intervals, effect sizes, degrees of freedom and P value noted<br>*Give P values as exact values whenever suitable.*
☒	☐	For Bayesian analysis, information on the choice of priors and Markov chain Monte Carlo settings
☒	☐	For hierarchical and complex designs, identification of the appropriate level for tests and full reporting of outcomes
☒	☐	Estimates of effect sizes (e.g. Cohen's d, Pearson's r), indicating how they were calculated

Our web collection on statistics for biologists contains articles on many of the points above.

Software and code

Policy information about availability of computer code

Data collection	ASTRA 7.3.2.19, ProteomeLab 6.04
Data analysis	XIA2 pipeline 0.5.9, Aimless 0.7.4, SHELX 2018/1, PHASER 2.8.3, PHENIX 1.14, REFMAC 5.8.0258, TLSMD, COOT 0.8.9.2, DIALS 1.14.5 Molprobity 4.2, PDB-REDO 7.35, Pymol 2.3.2, DALI, PDBePISA, tBLASTn, MAFFT 7.453, FastTree 2.1.11, ConSurf, ClustalW, ASTRA 7.3.2.19, SEDFIT 15.01, SEDPHAT 12.1, Image Lab 6.0.1

For manuscripts utilizing custom algorithms or software that are central to the research but not yet described in published literature, software must be made available to editors and reviewers. We strongly encourage code deposition in a community repository (e.g. GitHub). See the Nature Research guidelines for submitting code & software for further information.

Data

Policy information about availability of data

All manuscripts must include a data availability statement. This statement should provide the following information, where applicable:

- Accession codes, unique identifiers, or web links for publicly available datasets
- A list of figures that have associated raw data
- A description of any restrictions on data availability

For sequence conservation analysis, Saccharomyces spp genomes were obtained from SGD (https://www.yeastgenome.org/). Protein structures used in structural alignments were obtained from the Protein Data Bank (https://www.rcsb.org/). The atomic coordinates and structure factors for p18mAUG1, p18mAUG2 and p18mAUG2-A273V have been deposited in the Protein Data Bank under accession numbers 7NLH (https://doi.org/10.2210/pdb7NLH/pdb), 7NLI (https://doi.org/10.2210/pdb7NLI/pdb) and 7NLG (https://doi.org/10.2210/pdb7NLG/pdb). The entire p18m sequence alignment is available to download from the Figshare repository (https://crick.figshare.com/articles/dataset/Cottee_Supplementary_datafile1_Ty1p18_alignment_txt/15060366). The Source data and whole blot images underlying Figs 1d, 1f, 3b-c, 3f-h, 4b-c, 4e-f, 5c-d, 6a-g, and Supplementary Figs 6a-c, and 7 are provided as a Source Data file. All remaining data are contained

within the article.

Field-specific reporting

Please select the one below that is the best fit for your research. If you are not sure, read the appropriate sections before making your selection.

- [x] Life sciences
- [ ] Behavioural & social sciences
- [ ] Ecological, evolutionary & environmental sciences

For a reference copy of the document with all sections, see nature.com/documents/nr-reporting-summary-flat.pdf

Life sciences study design

All studies must disclose on these points even when the disclosure is negative.

Sample size	No sample-size calculations were performed. For quantitative measurement of Ty1 mobility, sample size was chosen based on 30 years of experience using the Ty1his3-AI retromobility assay, originally developed by the Garfinkel lab (citation 44). The application of appropriate statistical tests and methods detailed in publications from the Garfinkel lab (refer to citations 6, 10, 26, 28, 45, 53, and 64) validate the choice of sample size. Importantly, over 100,000 transposition events are sampled in each independent liquid culture expressing wild type Ty1his3-AI. This robust sensitivity, ranging over four logs, also permits quantitative reproducible detection of less frequent transposition events, such as those generated in the presence of the p18m restriction factor or by Gag mutants. Overall, the design described here is sufficient to detect meaningful biological differences with significant reproducibility.
Data exclusions	No data were excluded. All trials were successful.
Replication	Overall, data is representative of at least three biological replicates. For quantitative retromobility assays, data represents at least four independent galactose-inductions repeated three times. All replicates were successful.
Randomization	Randomization is built into all of our experiments in budding yeast in vivo as colonies are chosen randomly for analysis.
Blinding	Due to the nature of the experimental setup, blinding was not practical because the same investigator was doing group allocation during data collection and analysis. The corresponding authors also validated primary data collection and analysis.

Reporting for specific materials, systems and methods

We require information from authors about some types of materials, experimental systems and methods used in many studies. Here, indicate whether each material, system or method listed is relevant to your study. If you are not sure if a list item applies to your research, read the appropriate section before selecting a response.

Materials & experimental systems

n/a	Involved in the study	
☐	☒	Antibodies
☒	☐	Eukaryotic cell lines
☒	☐	Palaeontology and archaeology
☒	☐	Animals and other organisms
☒	☐	Human research participants
☒	☐	Clinical data
☒	☐	Dual use research of concern

Methods

n/a	Involved in the study	
☒	☐	ChIP-seq
☒	☐	Flow cytometry
☒	☐	MRI-based neuroimaging

Antibodies

Antibodies used	Antibodies used from commercial sources:<br>• mouse monoclonal anti-Pgk1 antibody clone 22C5D8 (Thermo Fisher Scientific Cat# 459250, RRID:AB_2532235; lot TA2506432)<br><br>• rabbit monoclonal anti-hexa-histidine antibody clone RM146 (Thermo Fisher Scientific Cat# MA5-33032, RRID:AB_2810125; lot VG3038162)<br><br>Previously generated custom antibodies used and references:<br>• mouse monoclonal anti-TY tag antibody clone BB2 (Bastin et al., 1996, PMID: 8813669)<br><br>• rabbit polyclonal anti-p18 antisera (Saha et al., 2015, PMID: 25609815)
Validation	Antibodies were validated by immunoblotting isogenic control yeast strains that lack the epitope:<br><br>The Pgk1 antibody is commercially available and validated by the manufacturer for use in Western blotting and immunocytochemistry in S. cerevisiae.

The hexa-histidine antibody is commercially available and validated by the manufacturer to specifically react with recombinant proteins containing the 6xHis-Tag or 10xHis-Tag fused to either the amino or carboxy terminus. No cross reactivity with other endogenous proteins in mammalian or bacteria cells is observed. It was further validated for Western blotting in Saccharomyces in the manuscript, for example Fig 1D.

The TY-tag antibody has been extensively verified previously, for example Saha et al., 2015, PMID: 25609815, Fig 11.

The anti-p18 antisera was validated by the original paper describing its generation: Saha et al., 2015, PMID: 25609815, Fig 3.
